# Transcriptome Analysis of Genes Responding to Infection of Leghorn Male Hepatocellular Cells With Fowl Adenovirus Serotype 4

**DOI:** 10.3389/fvets.2022.871038

**Published:** 2022-06-14

**Authors:** Xueping P. Wang, Bo Wen, Xiao J. Zhang, Lei Ma, Xiu L. Liang, Ming L. Zhang

**Affiliations:** ^1^Henan Joint International Research Laboratory of Veterinary Biologics Research and Application, Anyang Institute of Technology, Anyang, China; ^2^College of Veterinary Medicine, Northwest A&F University, Xianyang, China

**Keywords:** RNA sequencing, FAdV-4, LMHs, virus and host cell interaction, virus infection

## Abstract

Fowl adenovirus serotype 4 (FAdV-4) is a highly pathogenic virus with a broad host range that causes huge economic losses for the poultry industry worldwide. RNA sequencing has provided valuable and important mechanistic clues regarding FAdV-4–host interactions. However, the pathogenic mechanism and host's responses after FAdV-4 infection remains limited. In this study, we used transcriptome analysis to identify dynamic changes in differentially expressed genes (DEGs) at five characteristic stages (12, 24, 36, 48, and 60 h) post infection (hpi) with FAdV-4. A total of 8,242 DEGs were identified based on comparison of five infection stages: 0 and 12, 12 and 24, 24 and 36, 36 and 48, and 48 and 60 hpi. In addition, at these five important time points, we found 37 common upregulated or downregulated DEGs, suggesting a common role for these genes in host response to viral infection. The predicted function of these DEGs using Gene Ontology and Kyoto Encyclopedia of Genes and Genomes analyses revealed that these DEGs were associated with viral invasion, host metabolic pathways and host immunosuppression. Interestingly, genes involved in viral invasion, probably *EGR1, SOCS3*, and *THBS1*, were related to FAdV-4 infection. Validation of nine randomly selected DEGs using quantitative reverse-transcription PCR produced results that were highly consistent with those of RNA sequencing. This transcriptomic profiling provides valuable information for investigating the molecular mechanisms underlying host–FAdV-4 interactions. These data support the current molecular knowledge regarding FAdV-4 infection and chicken defense mechanisms.

## Introduction

Fowl adenovirus serotype 4 (FAdV-4) is a member of the *Aviadenovirus* genus, *Adenoviridae* family. FAdV-4 is a non-enveloped virus with a double-stranded DNA genome. It is ~43.7 kb in length and encodes 11 and 32 structural and non-structural proteins, respectively (1). FAdV-4 causes hydropericardium hepatitis syndrome (HHS), a severe disease of poultry worldwide, which is characterized by pericardial effusion and associated with high mortality rates (30 to 80%), resulting in major financial losses all over the world. The first outbreak of HHS was recorded in 1987 in Angara Goth, Pakistan. Since then, FAdV-4 has become prevalent in several other parts of the world (2–4). Prior to 2014, HHS was only sporadically reported in poultry in China. However, since 2015, a significant increase in the frequency of HHS outbreaks has been noted in various production areas of broilers in China, causing major economic burden (5–7).

Chicken has huge commercial value globally in the food industry (8). However, FAdV-4 infection is a significant threat to the chicken industry (9). Therefore, understanding the molecular response of FAdV-4 disease-resistance is vital for prevention of FAdV-4 infection. Important innate signaling pathways activated during the molecular response to infection may assist development of vaccines and anti-viral drugs (10–12). Leghorn male hepatocellular (LMH) cells belong to an immortalized chicken hepatic cell line and are the primary target cells of FAdV-4; LMH cells therefore play an important role in *in vitro* studies of host–FAdV-4 interactions (1, 13, 14). Previous research has attempted to elucidate the mechanism of FAdV-4–host interactions. These studies provide valuable and important mechanistic clues regarding virus–host interactions; however, our understanding of the FAdV-4–host cell interaction mechanism remains limited.

The aim of this study was to gain more comprehensive insight into the infection of host cells by FAdV-4. We used transcriptomics for the first time to our knowledge to investigate gene expression in LMH cells at five characteristic stages (12, 24, 36, 48, and 60 h) post infection (hpi) with FAdV-4. We then elucidated the molecular mechanisms (e.g., viral invasion, immunosuppression, and metabolic pathways) affected by viral infection. Our findings provide novel information regarding the mechanisms underlying virus–host interactions and the pathogenesis of FAdV-4.

## Materials and Methods

### Virus and Cells

FAdV-4 isolate SX17 (GenBank: MF592716.1) and LMH cells were kindly provided by Dr. Jing-Yu Wang (Northwest A&F University, China). LMH cells (4 × 10^5^ cells/well) were cultured in six-well plates maintained at 37°C in a 5% CO_2_ atmosphere in Dulbecco's modified Eagle's medium (DMEM, Gibco) with 10% fetal bovine serum (FBS). Cells were cultured for ~24 h to reach 80% confluence and subsequently infected with FAdV-4 (MOI = 0.1); uninfected LMH cells were used as a mock control. FAdV-4 infected with LMH Cells caused cytopathic effect (CPE), viral titers and viral loads as previously described (15).

### RNA Isolation, Library Construction, and RNA Sequencing (RNA-Seq)

TRIzol reagent (Invitrogen; USA) was used to isolate total RNA from 18 samples (five groups) according to the manufacturer's instructions. RNA purity and concentration were determined by ultraviolet absorbance using a Nanodrop Nano Photometer spectrophotometer (IMPLEN, USA), while RNA integrity was evaluated using an Agilent 2100 Bioanalyzer (Agilent Technologies, USA). Total RNA (3 μg) was used to construct a cDNA library using an RNA Library Prep Kit for Illumina (New England Biolabs, USA), according to the instructions provided by the manufacturer. In brief, total RNA was treated with DNaseI (Invitrogen, Life Technologies, USA) to remove genomic DNA contamination. This was followed by enrichment of poly(A) mRNA using oligo d(T) magnetic beads (Invitrogen). mRNA was next treated with fragmentation buffer, and cleaved RNA was copied into first-strand cDNA fragments using reverse transcriptase (Invitrogen) and random hexamer primers. Second-strand cDNA fragments were synthesized using Second Strand Synthesis Enzyme Mix, including DNA polymerase I, buffer, deoxyribonucleoside triphosphate, and RNase H. After purification and paired-end repair, cDNA fragments were ligated to sequencing adapters and amplified by PCR to obtain the final paired-end library. Libraries were subsequently sequenced using the Illumina HiSeq 4000 sequencing platform (Novogene, Shanghai, China).

### Transcriptome Data Analysis

To ensure high accuracy and reliability of the results of subsequent analyses, the original ordinal number was determined by filtering to obtain high-quality sequencing data (clean data). The original sequencing data were filtered to remove low-quality reads as follows: (1) removal of adapter sequences with a quality score < 20 and sequences with an N base rate of raw reads > 10%; and (2) removal of adapter and quality-trimmed short segments <25 bp in length. The remaining reads were termed “clean reads” and stored in FASTQ format. For mapping clean reads, Bowtie 2 was used to build reference genes and HISAT (hierarchical indexing for spliced alignment of transcripts) was used to reference the genome.

For gene expression analysis, gene expression levels were calculated and RSEM (RNA-Seq by Expectation-Maximization) was used to estimate the unigene FPKM value. Default criteria of | log2 fold-change | > 1 and false discovery rate < 0.05 denoted significant changes in differentially expressed genes (DEGs). Gene Ontology (GO), and pathway annotation and enrichment analyses were performed using the National Center for Biotechnology Information GO (http://www.geneontology.org) and Kyoto Encyclopedia of Genes and Genomes (KEGG) (http://www.genome.jp/kegg/) pathway databases. Cluster and Java Tree view software were used to conduct hierarchical cluster analysis of gene expression patterns. All gene expression data presented were obtained from three independent replicates. Original sequence data were submitted to the NCBI database (accession number: SUB9560975).

### Quantitative Reverse-Transcription PCR (qRT-PCR) to Validate Gene Expression

Nine genes were randomly selected for qRT-PCR analysis to validate the reproducibility and repeatability of the DEGs identified from transcriptome sequencing. Primer sequences used for qRT-PCR are listed in Supplementary Table 1. All primers were synthesized by Sangon Biotech (Shanghai, China). Total RNA was extracted from cell-line pellets using TRIzol reagent (Invitrogen, USA), according to the instructions provided by the manufacturer. RNA samples were reverse transcribed into cDNAs using a PrimeScript™ RT reagent Kit (TaKaRa, China). Relative gene expression values were calculated using the 2^−ΔΔCT^ method as previously described (16). mRNA levels were calculated with reference to those of the housekeeping gene β*-actin*. Each reaction was performed in triplicate.

## Results

### Growth Kinetics of FAdV-4-Infected LMH Cells

**Figure 1:**
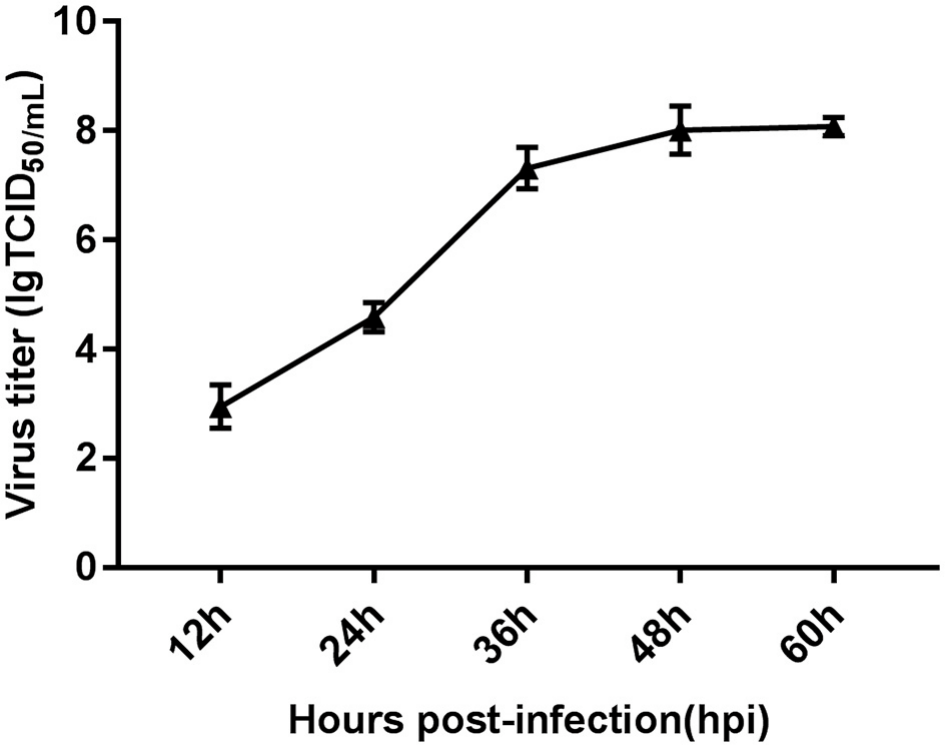
Characteristics of FAdV-4-infected LMH cells assessed *via* viral replication analysis. One-step growth curve of FAdV-4-infected LMH cells at a multiplicity of infection of 0.1, obtained using quantitative real-time PCR. Each time point represents the mean ± SEM of the *Hexon* gene from the FAdV-4 genome. SEM, standard error of the mean.

We determined the growth kinetics of FAdV-4 replication in primary LMH cells by monitoring the cytopathic effect and viral titers at 0, 12, 24, 36, 48, and 60 hpi. Viral load was observed at 12 hpi and increased slightly between 12 and 24 hpi. Subsequently, viral load increased significantly from 24 and 48 hpi; viral replication plateaued at 48 hpi (Figure 1). The wide range of infection time points used in this study allowed us to identify fluctuations in gene expression. This approach may enhance our understanding of the molecular response to infection with FAdV-4.

### RNA-Seq and Read Assembly

We used LMH cells at five time points (12, 24, 36, 48, and 60 hpi) after infection with FAdV-4 (each condition with three biological replicates); the data obtained at these time points were compared with those obtained at 0 hpi (three biological replicates of mock control). A total of 330.568 GB of clean data (sequencing data after quality control) were obtained. The average amount of clean data for each sample was 15.741 GB. The percentage of Q30 bases was > 92.28%, and the GC content ranged between 46.09 and 55.24%. Ribosomal RNA sequences were removed, and low-quality reads were discarded. We then mapped 18 high-quality reads to the chicken (*Gallus domesticus*) genome using HISAT (Table 1). The mean percentage of high-quality reads aligned to the reference genome was 84.96%. Qualifying sample data were evaluated according to multiple metrics. RNA sequencing raw data were deposited in the National Center for Biotechnology Information Gene Expression Omnibus (GEO) under accession number SRP149860.

**Table 1:** Statistics of the RNA-seq dataset.

**Sample**	**Raw reads**	**Raw bases**	**Q20%**	**Q30%**	**GC%**
Mock-1	93,753,712	14,063,056,800	97.86	94.09	46.18
Mock-2	10,793,405,6	16,190,108,400	97.53	93.52	46.73
Mock-3	94,306,864	141,460,296,00	97.65	93.90	46.59
12hpi-4	55,915,519	838,732,7850	97.82	94.27	47.52
12hpi-5	60,023,740	900,356,100,0	98.05	94.63	47.35
12hpi-6	11,935,448,6	179,031,729,00	97.67	93.83	47.64
24hpi-7	10,886,719,6	163,300,794,00	96.83	91.96	49.81
24hpi-8	10,271,892,6	154,078,389,00	97.65	93.82	48.60
24hpi-9	11,363,687,4	170,455,311,00	97.56	93.71	49.32
36hpi-10	12,564,059,6	188,460,894,00	97.36	93.60	52.54
36hpi-11	134,471,716	201,707,574,00	97.59	93.85	54.11
36hpi-12	123,828,996	185,743,494,00	97.80	94.28	54.12
48hpi-13	108,248,922	162,373,383,00	97.80	94.12	52.25
48hpi-14	112,423,808	168,635,712,00	97.54	93.79	52.31
48hpi-15	116,453,356	174,680,034,00	97.72	94.06	51.57
60hpi-16	117,541,138	17,631,170,700	97.85	94.35	54.81
60hpi-17	131,385,360	19,707,804,000	97.89	94.37	55.03
60hpi-18	130,098,414	19,514,762,100	97.78	94.15	55.23

### Identification of Differentially Expressed Transcripts

**Figure 2:**
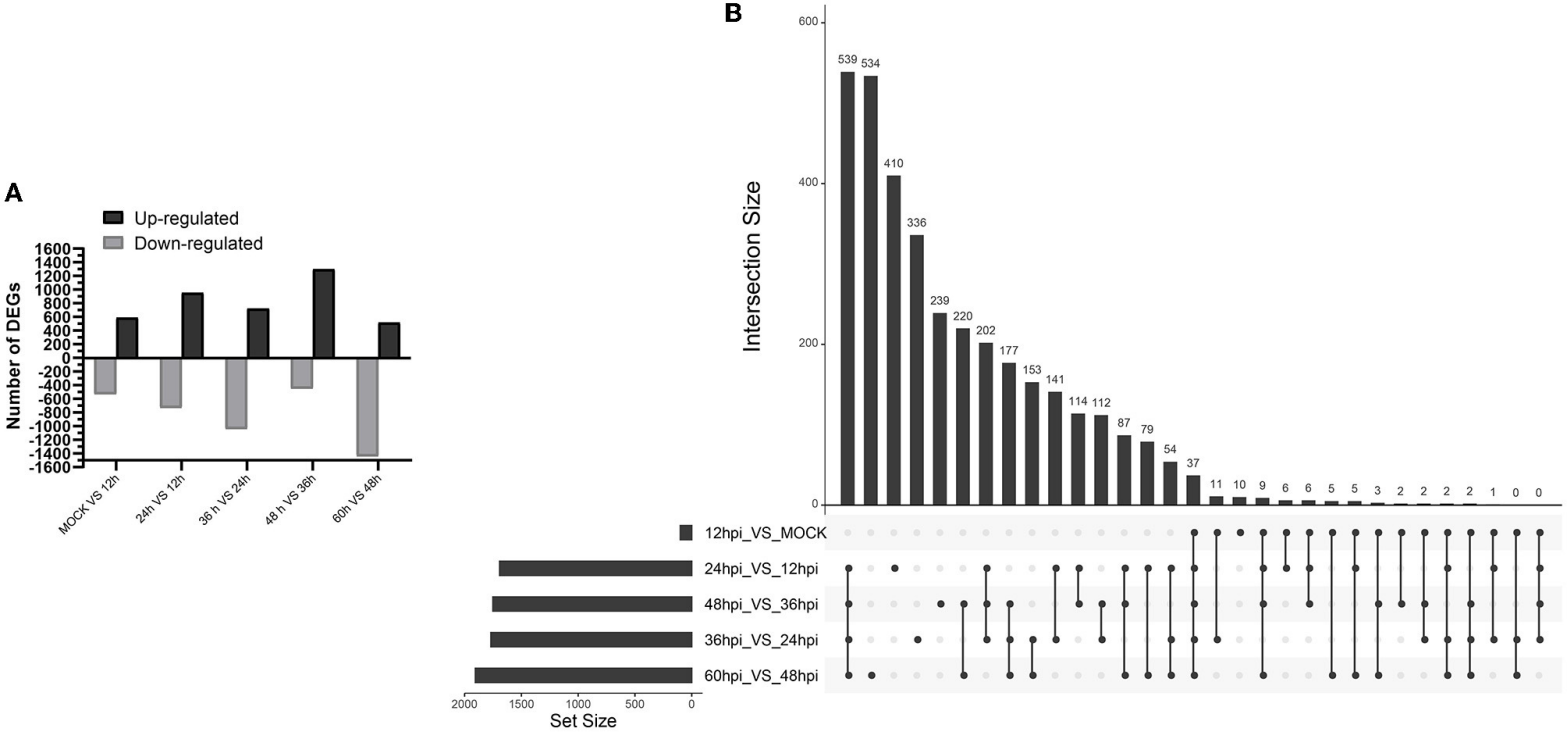
Identification and clustering of DEGs. **(A)** Number of differentially expressed genes at five time points after infection with FAdV-4. **(B)** Up Set plot showing unique and common DEGs in response to infection with FAdV-4 at five time points.

A total of 8,242 DEGs were identified at five important time points (12, 24, 36, 48, and 60 hpi) (Figure 2A). At 0 and 12, 12 and 24, 24 and 36, 36 and 48, and 48 and 60 hpi, we identified 589, 956, 724, 1,301, and 518 upregulated DEGs and 535, 736, 1,045, 450, and 1,442 downregulated DEGs (Supplementary Files 1–5). We identified 37 common upregulated and downregulated DEGs at all time points (Supplementary Files 11). Moreover, 10, 410, 336, 239, and 534 DEGs were unique to comparisons of 0 and 12, 12 and 24, 24 and 36, 36 and 48, and 48 and 60 hpi, respectively (Figure 2B).

Hierarchical clustering analysis revealed gradual changes in gene expression across at 0 and 12, 12 and 24, 24 and 36, 36 and 48, and 48 and 60 hpi; the left side of the hierarchical clustering gene tree shows further classification of DEGs (Supplementary Files 10).

### GO Enrichment Analyses of DEGs

**Figure 3:**
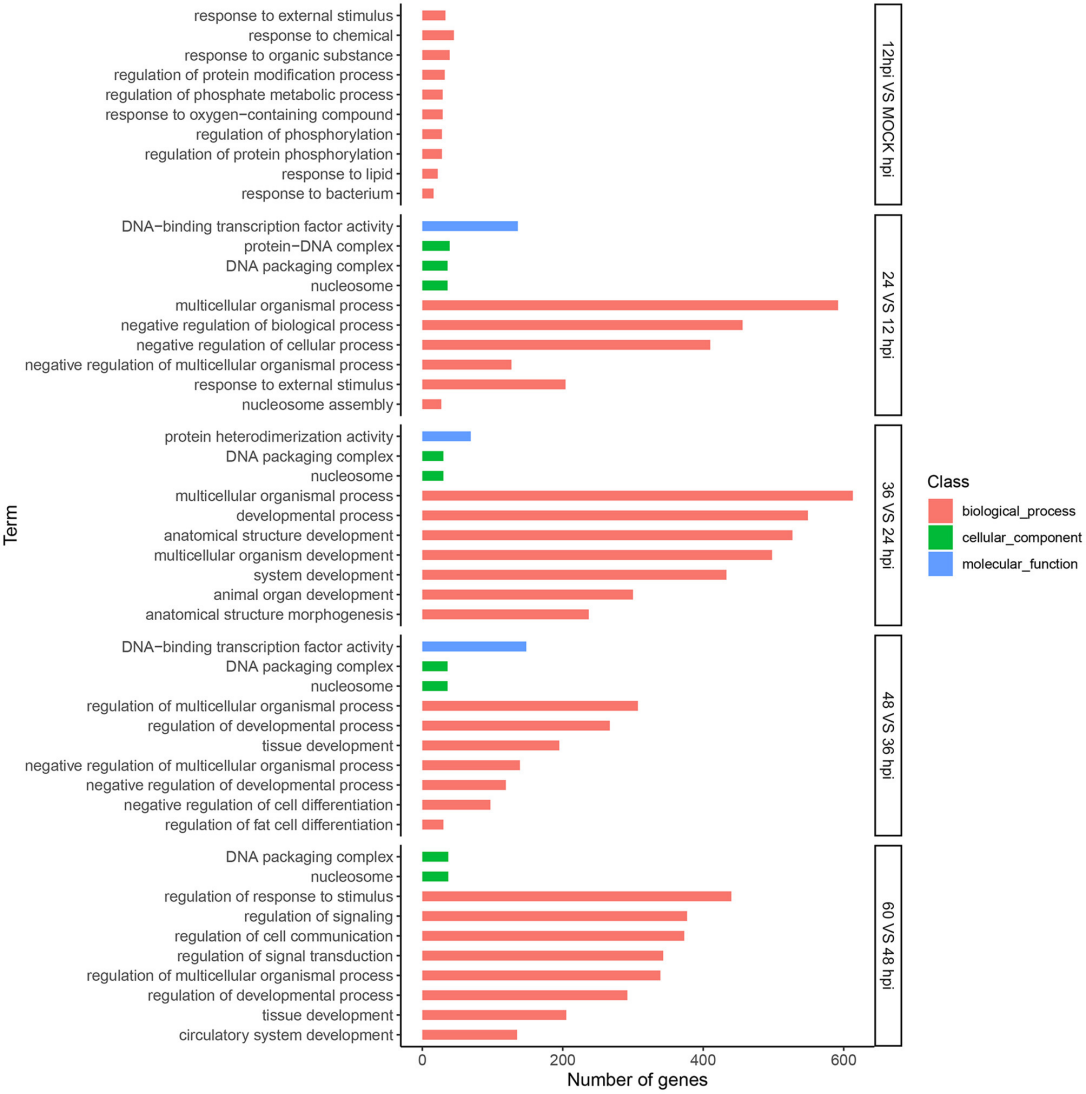
Analysis of DEGs using Gene Ontology (GO) terms. Top 10 GO categories significantly (*P*-value < 0.05) enriched in biological processes at five time points. The x-axis represents the number of genes, and the y-axis represents functional groups.

We conducted GO analysis to identify the biological processes involved in FAdV-4 infection. Among DEGs between 0 and 12 hpi, 8,348 unigenes were assigned GO terms in the cellular component (676), molecular function (1,347), and biological process (6,325) categories, respectively. For the 12 and 24 hpi comparison, 9,742 unigenes were assigned to 834, 1,651, and 7,257 GO terms in the aforementioned categories, respectively. For the 24 and 36 hpi comparison, 9,695 unigenes were assigned to 844, 1,681, and 7,170 GO terms in the aforementioned categories, respectively. For the 36 and 48 hpi comparison, 9,856 unigenes were assigned to 831, 1,636, and 7,389 GO terms in the aforementioned categories, respectively. Finally, for the 48 and 60 hpi comparison, 10,482 unigenes were assigned to 906, 1,707, and 7,869 GO terms in the aforementioned categories, respectively. We next selected the 10 most significant molecular functions and biological processes with altered gene expression among viral infection stages (Figure 3).

### KEGG Pathway Enrichment Analysis of DEGs

**Figure 4:**
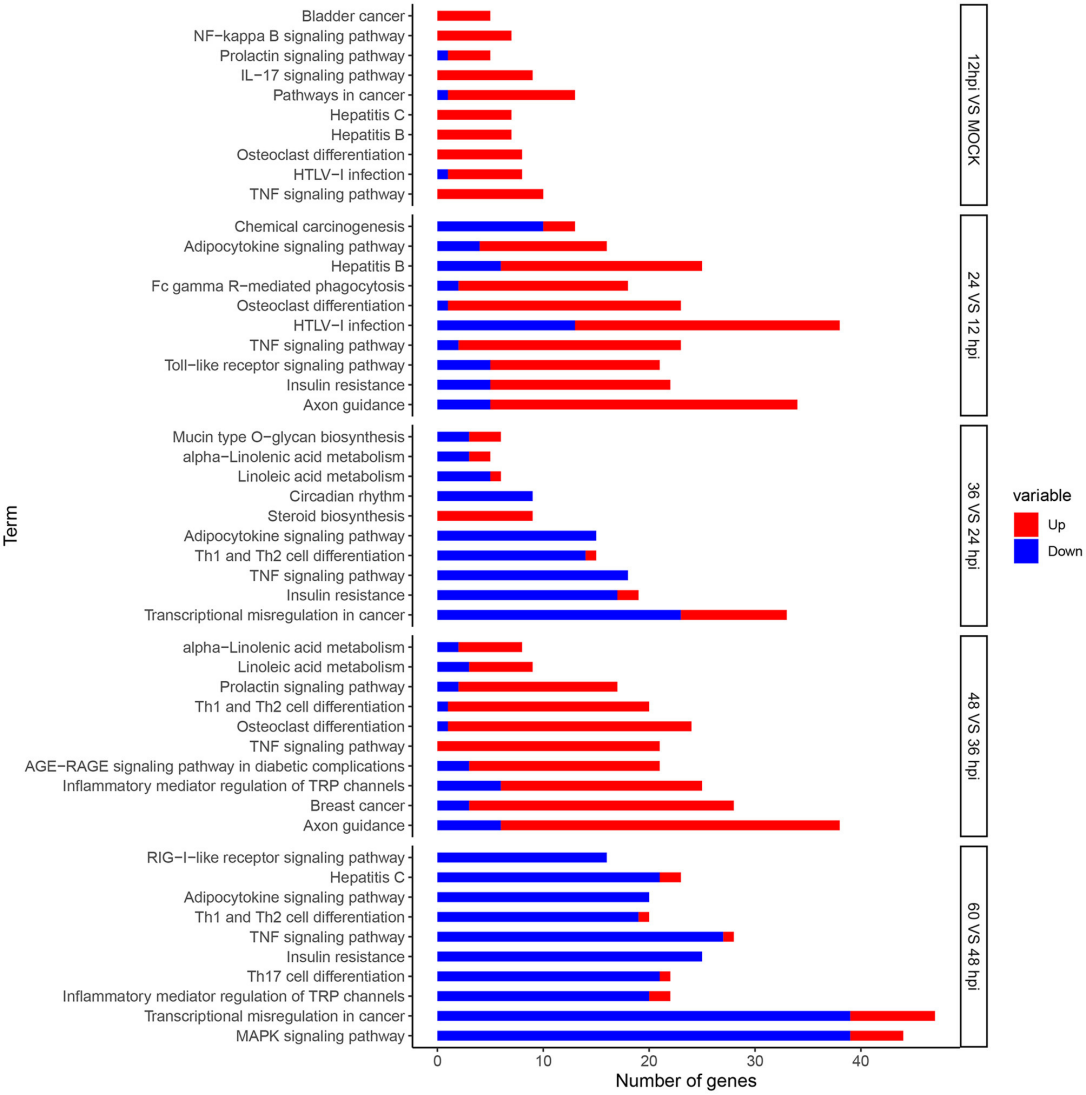
Analysis of DEGs using Kyoto Encyclopedia of Genes and Genomes (KEGG) terms. Top 10 KEGG pathways significantly (*P*-value < 0.05) enriched in biological processes at five time points. The x-axis represents the number of genes, and the y-axis represents functional groups. Red and blue portions of the bar represent the number of upregulated and downregulated genes, respectively.

Enrichment analysis of KEGG pathways can further identify signal transduction pathways in which DEGs participate. We mapped 8,242 DEGs to referential canonical pathways via KEGG database analysis. DEGs identified at five time points, 0 and 12 hpi (620 DEGs), 12 and 24 hpi (1,226 DEGs), 24 and 36 hpi (724 DEGs), 36 and 48 hpi (1,301 DEGs), and 48 and 60 (1,960 DEGs), were enriched to 138, 324, 321, 329, and 326 KEGG pathways, respectively. The 10 most enriched pathways were determined according to *P*-value < 0.05 (Figure 4). We identified many DEGs playing important roles related to viral invasion, immunosuppression, and metabolic pathways (Supplementary Files 6–8).

**Figure 5:**
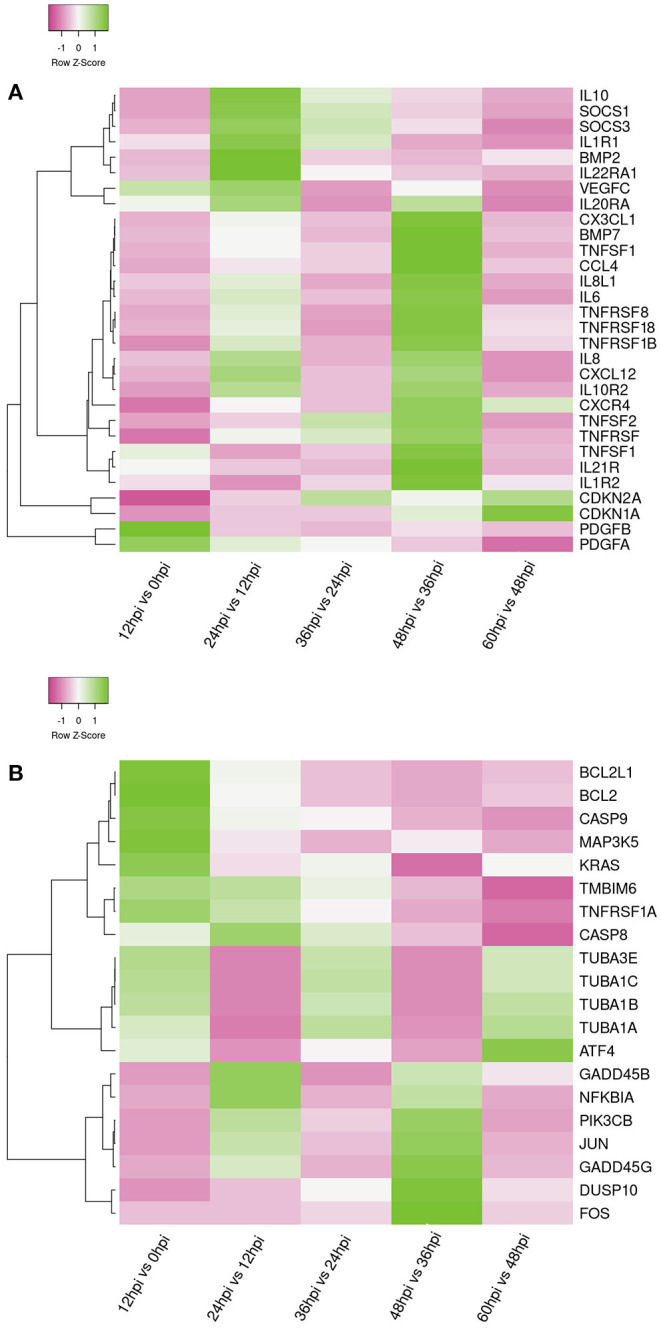
Heat maps shows the hierarchical clustering of genes involved in apoptosis genes **(A)** and Immune-related genes **(B)** at five time points. The color in the heat map represents the normalized isotig number. The color in the heat map represents gene expression changes (green, high gene expression; pink, low gene expression; white, no gene expression). DEGs are labeled on the left of the heat-map line.

In order to further analyze the differential expression host immunosuppression genes response during FAdV-4 infection. KEGG enrichment analyze, the DEGs were significantly enriched in immune-related signaling pathways, such as, TNF signaling pathway, IL-17 signaling pathway, Apoptosis, P13K-Akt signaling pathway, cytokine-cytokine receptor interaction, Th17 cell differentiation, Th1 and Th2 cell differentiation, Toll-like receptor signaling path way (Supplementary Files 9A–E). Meanwhile, Apoptosis and immune-related signaling pathway the changes in important gene expression shown by the heatmap (Figures 5A,B).

### Validation of RNA-Seq Data Using qRT-PCR

**Figure 6:**
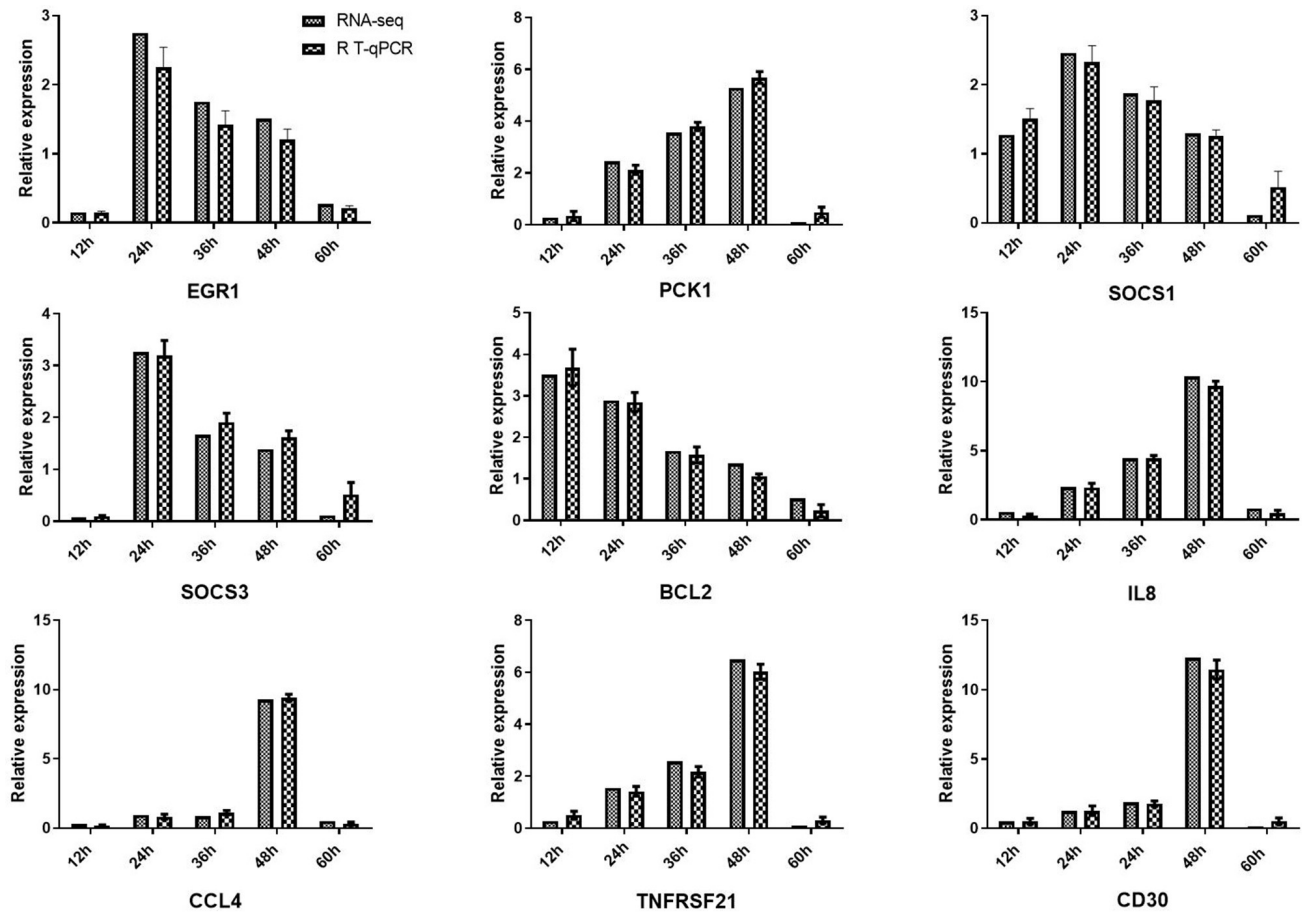
Validation of RNA-seq results using quantitative reverse-transcription PCR (qRT-PCR). Expression profiles of nine DEGs playing important roles in viral defense. qRT-PCR verified that mRNA levels of these genes were increased after viral infection, consistent with the altered expression profiles observed in transcriptome data. β*-actin* was used for normalization. The x-axis represents the different time points, and the y-axis represents the normalized fold-change of transcripts.

To further evaluate our library of differentially expressed unigenes, we used qRT-PCR to detect the expression of nine pro-inflammatory response genes (*early growth response 1* [*EGR1*], *suppressor of cytokine signaling 3* [*SOCS3*], *thrombospondin 1* [*THBS1*], *interleukin-8* [*IL8*], *suppressor of cytokine signaling 1* [*SOCS1*], *TNF receptor superfamily member 8* [*CD30*], *C-C motif chemokine ligand 4* [*CCL4*], *phosphoenolpyruvate carboxykinase1* [*PCK1*], *Apoptosis regulator proteins Bcl-2* [Bcl-2] and *TNF receptor superfamily member 21* [*TNFRSF21*] (Figure 6). Comparison of the fold-change in expression of the selected genes observed by RNA-seq and qRT-PCR showed that the two data sets were largely consistent.

## Discussion

First emerging in 2015 in China, cases of fowl adenovirus serotype 4 (FAdV-4) infection in broilers, laying hens, ducks, and geese have caused economic and social losses globally, including in India, South Korea, Hungary, Japan, Europe, and China (17, 18). As a recently discovered adenovirus, research on FAdV-4 remains insufficient. In this study, we employed transcriptome sequencing technology to explore crucial factors affecting host–virus interaction at five characteristic stages (12, 24, 36, 48, and 60 hpi) of FAdV-4 infection in LMH cells. Many important genes associated with viral invasion, host immunosuppression, and host metabolic pathways were differentially expressed.

When FAdV-4 infects a host, a complex battle between host cells and the virus takes place. Continuous replication of the virus damages host cells to different degrees and alters their physiological functions, leading to differential gene expression. DEGs identified following FAdV-4 infection of LMH cells can be divided into five infection periods based on their differential expression patterns. At the first stage of infection (0 to 12 hpi), we detected 1,124 DEGs between 0 and 12 hpi. At 12 hpi, entry of FAdV-4 into LMH cells induces a strong immune response, and host cells enter a static phase of balanced offensive and defensive attacks (19, 20). The second stage of infection between 12 and 24 hpi is a crucial time point for FAdV-4 and host cell interaction; beyond 24 hpi, the pathogen prevails, resulting in marked viral replication. We identified 1,692 DEGs between 12 and 24 hpi (20). These results were similar to those observed in previous studies (19). In the third stage of infection (24 to 36 hpi), we detected 1,769 DEGs between 24 and 36 hpi; during this time, the virus controls the metabolic machinery of host cells to produce more progeny virus. In the fourth stage of infection (36 and 48 hpi), we detected 1,751 DEGs between 48 and 36 hpi; among these different genes, expression of 1,301 DEGs increased dramatically while 450 DEGs were downregulated. The peak of FAdV-4 DNA replication occurs at 48 hpi and affects the host cell cycle, antiviral response, and cell proliferation as the virus changes its strategy from reproduction to efficient release and spread of progeny through destruction of the cell. In the late stage of virus infection (48 to 60 hpi), we detected 1,906 DEGs between 48 and 60 hpi; during this time, the number of DEGs was significantly increased compared with earlier stages of infection, with the majority being downregulated at 60 hpi compared with 48 hpi. This downregulation was non-specific and a consequence of imminent cell death. At this stage, FAdV-4 changes its strategy from reproduction to release of viral progeny and spread to adjacent cells through destruction of the cell, leading to gradual destruction of infected tissue.

From these five characteristic stages of FAdV-4 infection of host cells, we determined that regulation of host cell gene expression by FAdV-4 is specific, with FAdV-4 invading host cells to replicate its genome, produce new infectious virions, and spread virions to adjacent cells. These results are consistent with findings from previous studies on human adenovirus (19, 20). In addition, at these five important time points, we found 37 common differentially upregulated or downregulated genes, Among these common differential gene, the IL-8,SOCS3, NFKBIA, CCL4, NFAIP3, and TNIP2, which are associated with immune signaling pathways. Especially, TNFAIP3 belong to tumor necrosis factor were influenced during FAdV-4 infection process and play vital roles in the inflammatory response (21, 22). However, IL-8 uncontrolled expression can cause several inflammatory responses infected tissues and ultimately lead to death (23, 24). These results indicated that these common upregulated or downregulated genes play a common role for these genes in host response to viral infection. Importantly, our study covered a wide range of time points of LMH cell infection with FAdV-4, allowing us to identify a large number of DEGs relevant to viral invasion, immunity response, apoptosis, and metabolic pathways. In-depth analysis of these pathways will help us understand the mechanisms involved in FAdV-4–host interaction.

### Viral Invasion

Viral entry into cells is an extremely complex process involving multiple signaling pathways and receptor proteins. It is the premise for virus replication to cause pathological changes and consequently induce disease. During adenovirus infection, viral invasion occurs through adhesion to the cell surface and binding to host receptors, followed by membrane fusion and viral entry into the cell (20). In the early stages of FAdV-4 infection, we found that several important DEGs (e.g., THBS1, EGR1, and SOCS3) were upregulated. As previously reported, THBS1 plays a key role in pathophysiology of hantavirus infection (25, 26). EGR1 facilitates the replication of enterovirus 71 by directly targeting viral genomic RNA (27). The mitogen-activated protein kinase (MEK) signaling pathway can activate EGR1 during Foot-and-mouth disease infection (28). An important finding of the current and previous studies is that SOCS proteins are upregulated following viral infection; expression of *SOCS1* or *SOCS3* following viral infection promotes the replication of multiple viruses (29, 30). Further studies are warranted to clarify the main role and mechanism of SOCS protein following FADV-4 infection. Based on these observations, THBS1, EGR1, and SOCS3 are a good candidate gene for further functional studies.

KEGG analysis revealed that DEGs at 12 and 24 hpi were mainly enriched in the regulatory signaling pathways of receptor binding, including cell adhesion molecules, phosphatidylinositol 3-kinase-AKT (PI3K-AKT) pathway, toll-like receptor pathway, and endocytosis pathway, which are closely related to adenovirus infection (8, 19, 20). Previous studies have shown that cell adhesion molecules, as important cellular receptors, help numerous other viruses (e.g., human adenoviruses) to attach to and enter into target cells (31). The PI3K-AKT signaling pathway (PIK3AP1, PIK3CB, and PIK3R5) is closely related to adenovirus infection and specifically involves mediation of PI3K activation (32). The toll-like receptor signaling pathway can recognize viral nucleic acids participating in FAdV-4 infection (33, 34). The endocytosis pathway plays a critical role in mediation of different receptors during adenoviruses entry into host cells (35, 36).

Therefore, further investigation the role of candidate genes and signal pathways may play essential roles in the regulation of FAdV-4 infection, will not only provide new molecular targets for the development of antiviral drugs but also contribute to research vaccines in poultry.

### Metabolic Pathways

As strictly intracellular parasites, viruses depend on manipulation of the host metabolism to obtain the necessary energy and nutrients for their own survival and proliferation. Considerable evidence has shown that viral infection (e.g., adenovirus 5, influenza A virus, Newcastle disease virus, and herpes simplex virus-1) alters the metabolism of host cells (37–40). Different viruses require unique metabolic changes to successfully spread their progeny (41). In this study, we identified numerous significantly altered DEGs, which were enriched in important metabolism pathways, including carbohydrate metabolism, amino acids, lipid metabolism, and biosynthesis of secondary metabolites (42) (Supplementary File 6).

Carbohydrate metabolism is the primary carbon and energy source and plays a vital role in mammalian cells during homeostasis. Numerous metabolomic studies have identified an increase in carbohydrate metabolism caused by viral infection (39). For example, adenovirus can promote anabolic glucose metabolism in host cells and viral replication through activation of early region 4 open reading frame 1 (E4ORF1)-induced MYC. Studies of duck hepatitis A virus genotype 3 also demonstrated that glucose metabolism is a crucial hub during virus infection (43). In our study, genes associated with the glycolysis metabolic pathway, including *PCK1, phosphofructokinase, muscle* (*PFKM*), and *alcohol dehydrogenase* 1C (class I), gamma polypeptide (*ADH1C*), were upregulated at 12 and 48 hpi. Subsequently, during the rapid replication period of FAdV-4, expression levels of genes encoding various carbohydrate branch metabolites involved in fructose and mannose metabolism, glycosaminoglycan biosynthesis, tricarboxylic acid cycle, and the pentose phosphate pathway were significantly increased (44).

Amino acids are the most basic building blocks of living organisms. They serve as the essential substrate for the biosynthesis of many important biomolecules. In addition, amino acids can provide intermediate metabolites for glycolysis and the tricarboxylic acid cycle (45). Previous studies suggested that infection of human breast and bronchial epithelial cells with wild-type adenovirus 5 can increase glucose consumption. Additionally, adenovirus infection can selectively increase the expression of asparagine synthetase, which regulates the replication of adenovirus (46). Recent studies have found that FAdV-4 influences the metabolism of arginine to promote its replication (47). In the present study, KEGG enrichment analysis showed that amino acid metabolism pathways were upregulated during FAdV-4 infection; moreover, the production of arginine, glycine, serine, threonine, tyrosine, valine, leucine, isoleucine, alanine, aspartate, glutamate, cysteine, and methionine was increased during infection with FAdV-4. Collectively, these finding suggest that amino acid metabolism may contribute to FAdV-4 replication and deserves attention in future research studies.

Lipid is an important component of human cell tissue and a key structural component of the cell membrane, which plays a crucial role in a variety of biological processes. Numerous studies have demonstrated that viruses can take advantage of several core lipid metabolic pathways to successfully adhere to and invade host cells (38, 48). In the present study, genes involved in glycerol lipid metabolism, ether lipid metabolism, and sphingolipid metabolism were upregulated by FAdV-4 infection (Supplementary File 6).

In addition to the three major metabolic processes of host cells described above, we found that steroid biosynthesis and nitrogen metabolism were also altered after infection with FAdV-4. These are important host metabolic pathways that control the process and outcome of virus infections (49, 50). Further studies on metabolic genes and signaling pathways associated with FAdV-4 infection may provide some reference value for studying the pathogenesis and new diagnostic methods of FAdV-4.

### Immunosuppression

FAdV-4 is the primary pathogen of HHS, which has an immunosuppressive potential (51, 52). Previous studies have shown that apoptosis and inflammatory signaling activation are potent inducers of immunosuppressive mechanisms (4, 53). Interestingly, in this study, we observed that a substantial number of DEGs were enriched in the apoptosis-related and inflammatory pathways.

Apoptosis is a physiological and pathological phenomenon. Excessive apoptosis can lead to physical dysfunction, which results in a bystander effect of induced immunosuppression (4, 54). In the present study, we observed that infection of LMH cells with FAdV-4 activated a number of DEGs that regulate the apoptosis, apoptosis-fly, and apoptosis-multiple species signaling pathways, mainly containing pro-survival genes e.g., *TMBIM6 (Bax inhibitor), BCL2L1 (Bcl-xL), BCL2 apoptosis regulator* (*BCL2*); pro-apoptotic genes e.g., *caspase 8* (*CASP8*), *caspase 9* (*CASP*9), transcription factor subunit (FOS), Jun proto-oncogene, AP-1 transcription factor subunit (JUN), tubulin alpha 1a (TUBA1A), TUBA1B, TUBA1C, and TUBA3E (Figure 5A). CASP8 and CASP9 are the indicator of the mitochondrion-mediated apoptosis pathway. Once activated, this gene triggers characteristic apoptotic changes in cells (4, 55). Interestingly, we observed that BCL2 and BCL2 associated X, apoptosis regulator (BAX) were released. The BCL2 dimer regulates apoptosis by combining with BAX to form a heterodimer. This process leads to loss of the inhibitory effect of BCL2 on apoptosis, maintaining the stability of the host internal environment (56, 57). Furthermore, JUN and FOS regulate the gene encoding the Fas cell surface death receptor (FAS) ligand and play an important role as potent inducers of apoptosis (58–60).

A moderate inflammatory response can maintain immune system homeostasis (61, 62). However, persistent infection with a pathogenic microorganism may induce severe inflammation. In this study, infection of LMH cells with FAdV-4 activated numerous immune signaling pathways (Supplementary File 7). Our results indicated that these immune pathways were associated with several common DEGs, mainly those encoding pro-inflammatory genes e.g., IL1, IL-6, IL-8, IL-15, C-X3-C motif chemokine ligand 1 (CX3CL1), C-X-C motif chemokine receptor 4 (CXCR4), C-X-C motif chemokine ligand 12 (CXCL12), TNF superfamily member 10 (TNFSF10), TNFSF11, TNFSF13B, TNFSF18, TNFSF21 and anti-inflammatory genes e.g., SOCS3, CDKN1A, IL10, CDKN2A (Figure 5B). IL-6 and IL-8 are the most important pro-inflammatory cytokines in FAdV-4 infection (25). IL21R belongs to the Th17 cell differentiation pathway; this is a newly discovered subset of effector T helper cells, which mainly produce cytokines. Previous studies have demonstrated that IL21R is crucial for the differentiation of native T cells into Th17 cells and also plays a key role in the development of autoimmune disease (63). The Th1 and Th2 cell differentiation pathway was also activated, as evidenced by the upregulation of genes encoding cytokine-secreting Th1 (pro-inflammatory) cytokines (IL-17, IL-2). Th1 participates in cellular immunity, delays hypersensitivity inflammatory response, and plays a key role in host resistance to intracellular bacteria and other pathogens (64). TNFRSF13B and TNFSF21, members of the tumor necrosis factor receptor family, affect multiple cytokine pathways. They play an important role in initiating signal transduction and participate in complex immune responses, pathological injury, and the mediation of immunosuppression (61, 65, 66). We observed that, in LMH cells infected with FAdV-4, TNFRSF13B was downregulated, whereas TNFSF21 was upregulated. These findings are consistent with data obtained from studies of the above immune-related diseases (34, 61).

Altogether, the balance between these pro-survival genes, pro-apoptotic genes, pro-inflammatory and anti-inflammatory factors may be play important role for FAdV-4 immunosuppression. Further explore those of genes were enriched in the apoptosis-related and inflammatory pathways is essential to clarify the pathogenesis mechanisms of avian adenovirus and identify potential therapeutic strategies.

## Conclusion

This transcriptomic study offers novel insights for better understanding the response of LMH cells to infection with FAdV-4. Our data highlight the mechanisms involved in FAdV-4 invasion and FAdV-4-induced immunosuppression, as well as the effects of metabolic pathways on FAdV-4 infection. The basic data obtained in this study indicate the complexity of host–FAdV-4 interaction and provide new ideas for the development of effective vaccines against FAdV-4.

## Data Availability Statement

The datasets presented in this study can be found in online repositories. The names of the repository/repositories and accession number(s) can be found in the article/Supplementary Material.

## Author Contributions

XW conceived and designed the experiments and wrote sections of the manuscript. XW and BW performed the experiments. XZ, LM, and XL analyzed the data. MZ contributed reagents, materials, and analysis tools. All authors contributed to manuscript revision, read, and approved the submitted version.

## Funding

This work was supported by the National Natural Science Foundation of China [Grant No. NSFC32002313] and the Key Scientific Research Project of Henan Higher Education Institutions of China [Grant No. 20B230001].

## Conflict of Interest

The authors declare that the research was conducted in the absence of any commercial or financial relationships that could be construed as a potential conflict of interest.

## Publisher's Note

All claims expressed in this article are solely those of the authors and do not necessarily represent those of their affiliated organizations, or those of the publisher, the editors and the reviewers. Any product that may be evaluated in this article, or claim that may be made by its manufacturer, is not guaranteed or endorsed by the publisher.
