# Supplementary figures and images for: Transcriptome Analysis of Genes Responding to Infection of Leghorn Male Hepatocellular Cells With Fowl Adenovirus Serotype 4
Source: Front Vet Sci. 2022 Jun 14;9:871038. doi: 10.3389/fvets.2022.871038 (PMC9237548; doi:10.3389/fvets.2022.871038)

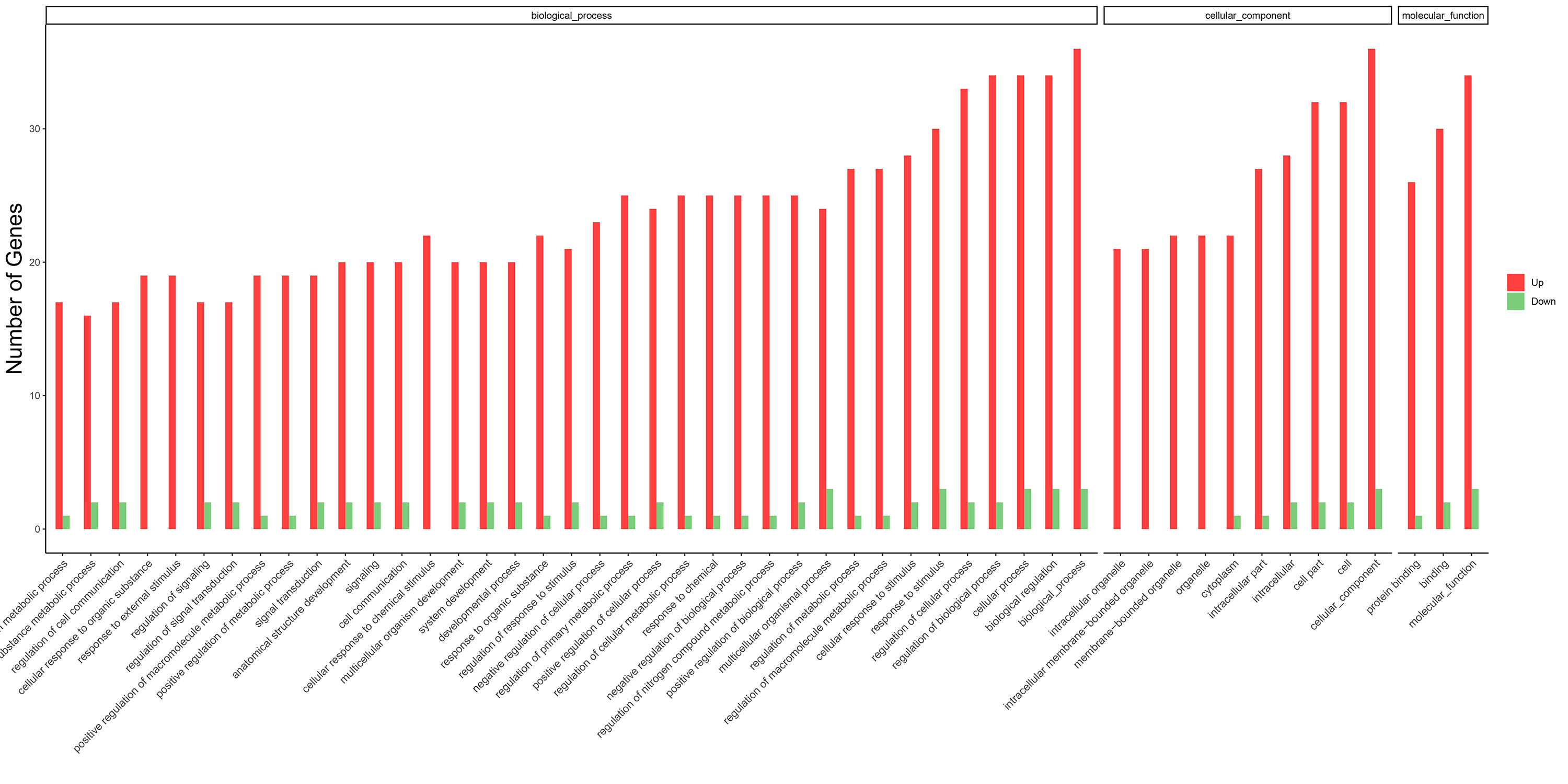

Supplement: Supplementary file 1 [file Image_1.TIF]

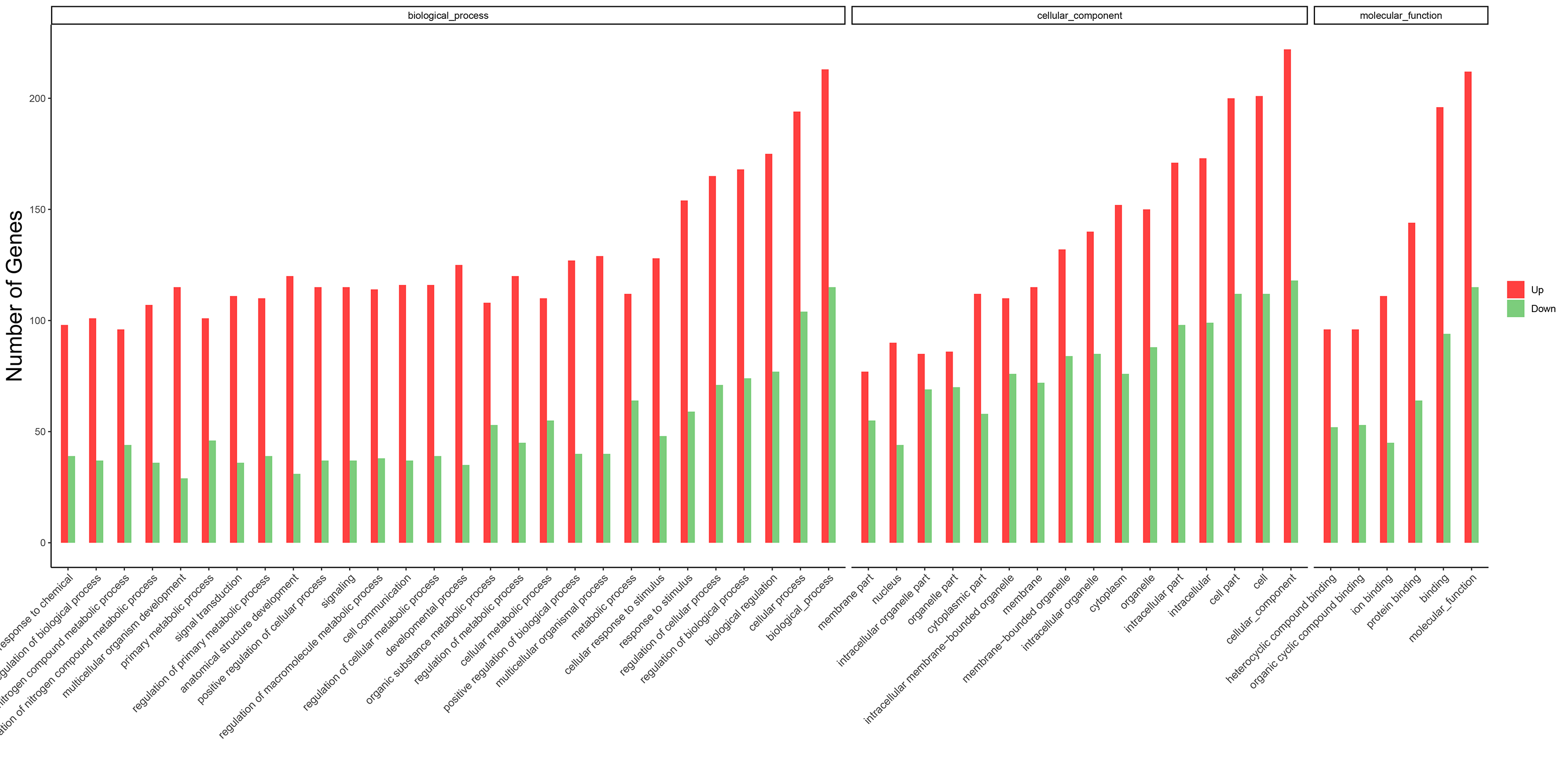

Supplement: Supplementary file 2 [file Image_2.TIF]

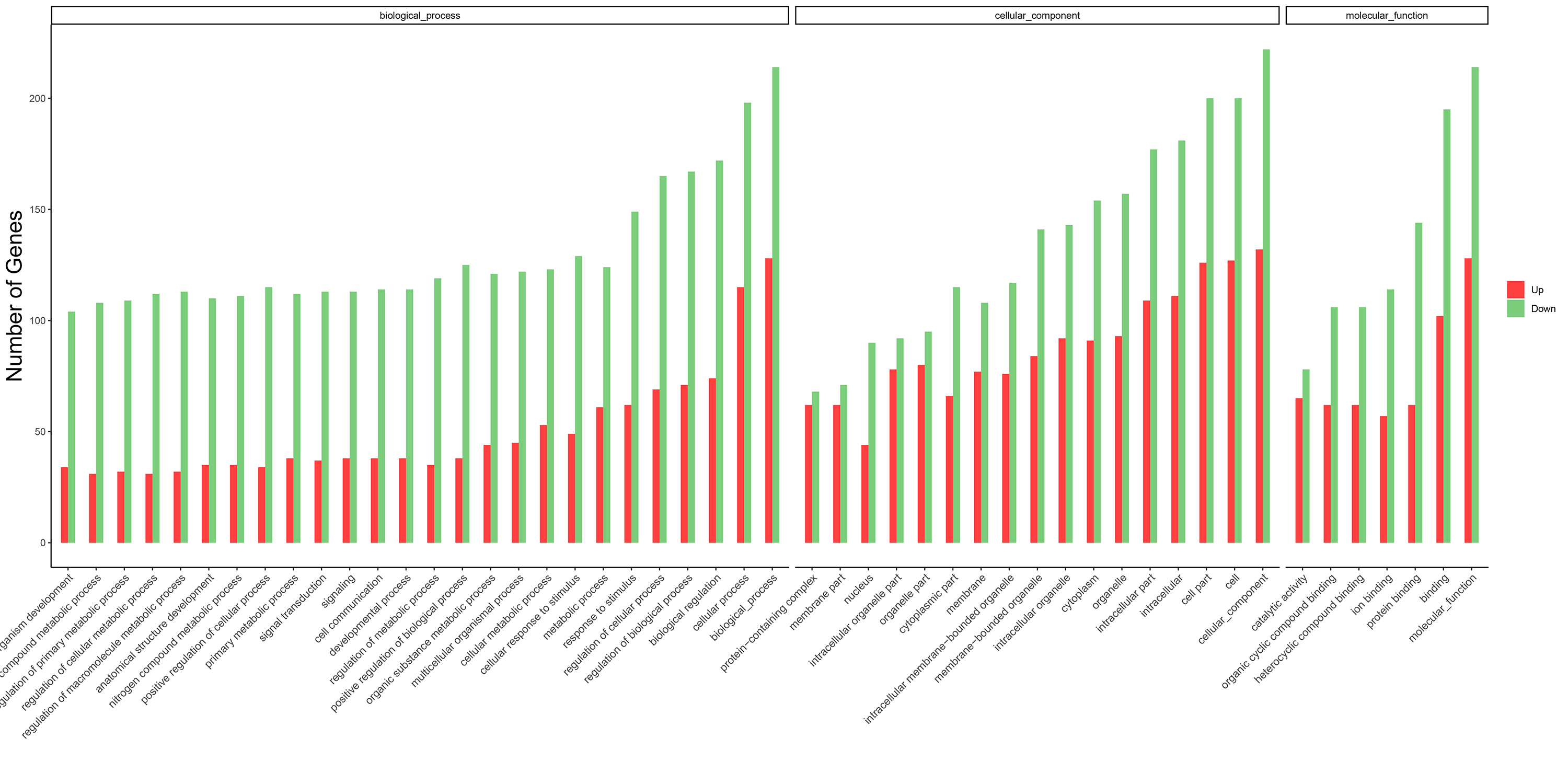

Supplement: Supplementary file 3 [file Image_3.TIF]

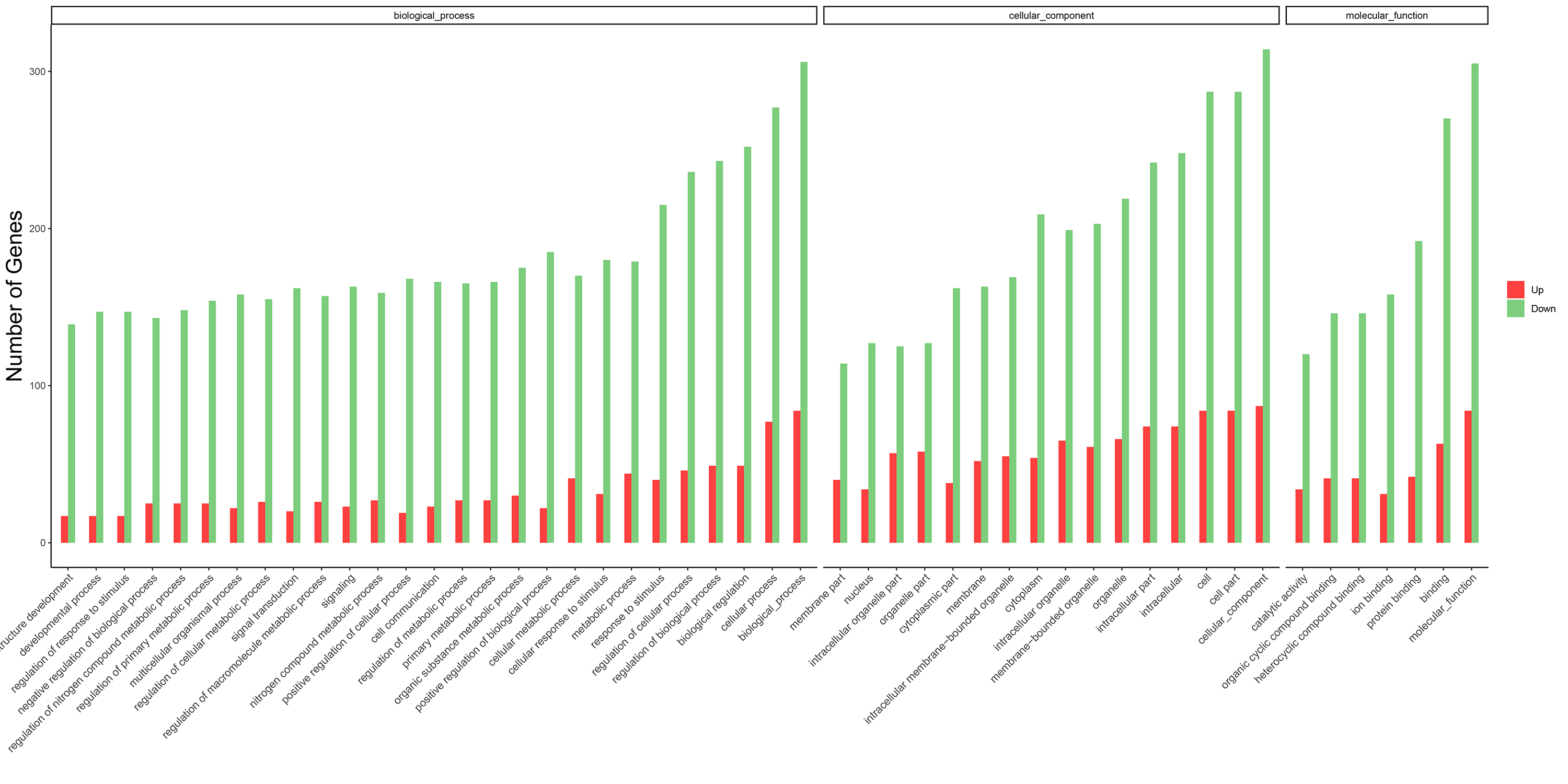

Supplement: Supplementary file 4 [file Image_4.TIF]

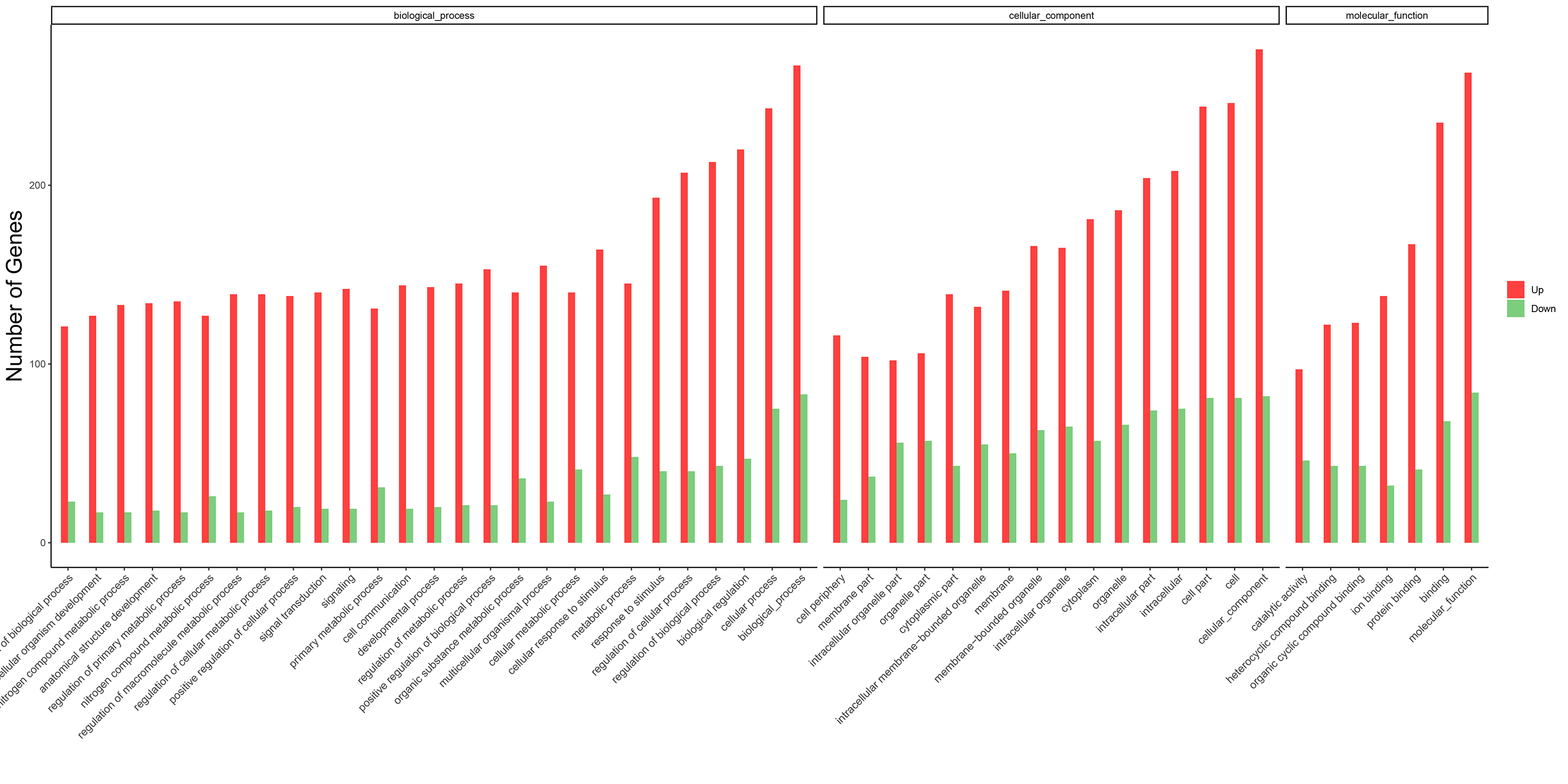

Supplement: Supplementary file 5 [file Image_5.TIF]

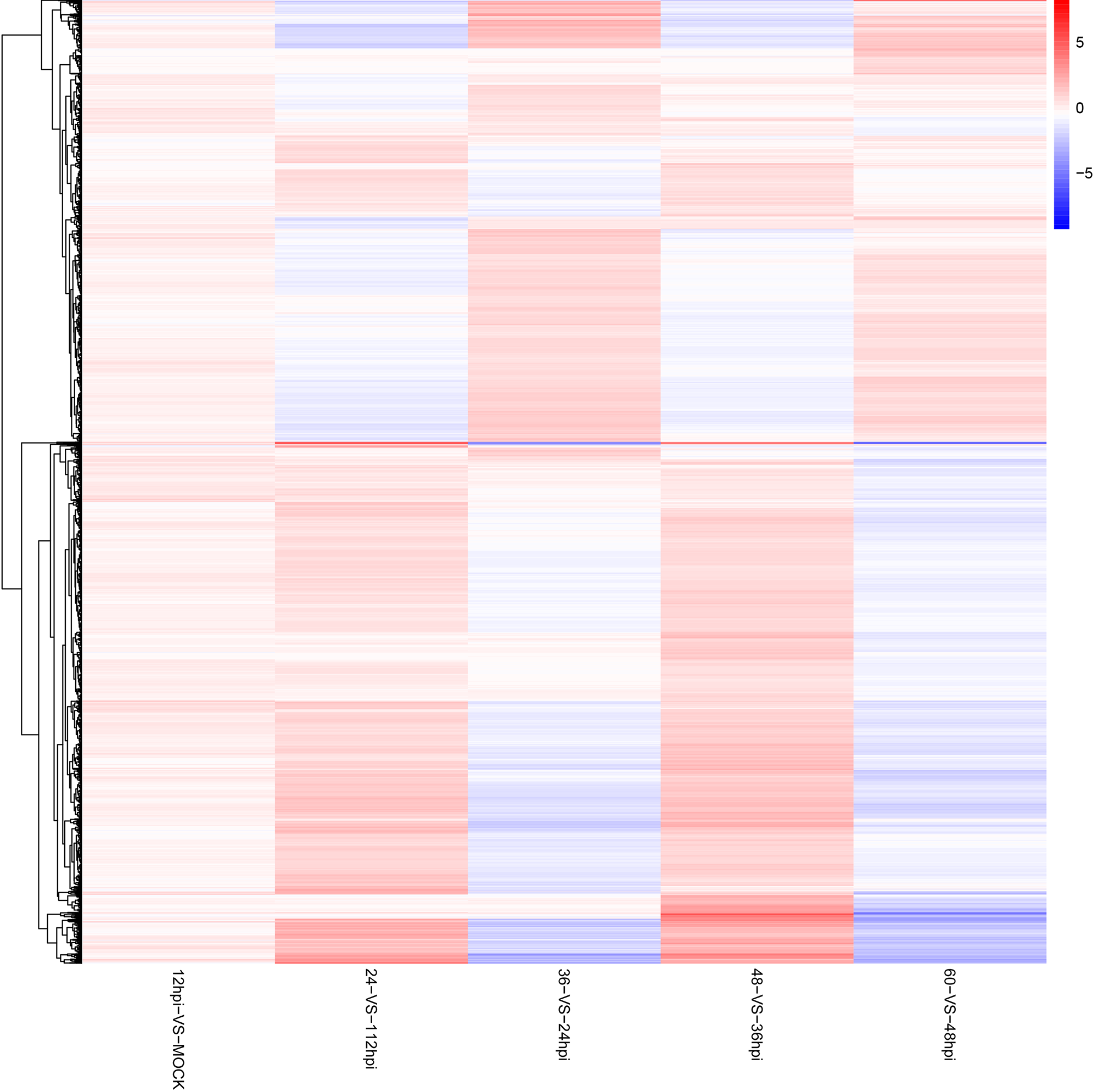

Supplement: Supplementary file 6 [file Image_6.TIF]
