# Supplementary material for: Transcriptome Analysis of Genes Responding to Infection of Leghorn Male Hepatocellular Cells With Fowl Adenovirus Serotype 4
Source: Front Vet Sci. 2022 Jun 14;9:871038. doi: 10.3389/fvets.2022.871038 (PMC9237548; doi:10.3389/fvets.2022.871038)
Supplement: Supplementary file 12 [file Table_6.DOCX]

Differentially expressed gene (DEGs) involved in viral invasion to FAdV-4 in LMH cells at 12,24,36,48,60hpi.

| Biological process | Gene symbol | Gene description | Source of DEGs |
| --- | --- | --- | --- |
| Toll-like receptor signaling pathway | CD40 | Clusters of differentiation 40 | 0hpi vs 12 hpi |
|  | CD80 | Clusters of differentiation 80 | 0hpi vs 12 hpi |
|  | FOS | Fos Proto-Oncogene, AP-1 Transcription Factor Subunit | 0hpi vs 12 hpi |
|  | IRF7 | Interferon regulatory factor 7 | 0hpi vs 12 hpi |
|  | PIK3CB | Phosphatidylinositol-4,5-Bisphosphate 3-Kinase Catalytic Subunit Beta | 0hpi vs 12 hpi |
|  | PIK3R1 | Phosphoinositide-3-Kinase Regulatory Subunit 1 | 0hpi vs 12 hpi |
|  | TLR2-1 | expression of the type1isoform of TLR2 | 0hpi vs 12 hpi |
|  | TLR5 | Toll-Like Receptor 5 | 0hpi vs 12 hpi |
|  | FADD | Fas Associated Via Death Domain | 12hpi vs 24 hpi |
|  | IL-12B | Interleukin 12B | 12hpi vs 24 hpi |
|  | IL8 | Interleukin-8 | 12hpi vs 24 hpi |
|  | IRF7 | Interferon regulatory factor 7 | 12hpi vs 24 hpi |
|  | JUN | Jun Proto-Oncogene, AP-1 Transcription Factor Subunit | 12hpi vs 24 hpi |
|  | MAP3K8 | Mitogen-Activated Protein Kinase Kinase Kinase 8 | 12hpi vs 24 hpi |
|  | MAPK10 | Mitogen-Activated Protein Kinase 10 | 12hpi vs 24 hpi |
|  | NFKB1 | Nuclear Factor Kappa B Subunit 1 | 12hpi vs 24 hpi |
|  | NFKBIA | NFKB Inhibitor Alpha | 12hpi vs 24 hpi |
|  | SPP1 | Secreted Phosphoprotein 1 | 12hpi vs 24 hpi |
|  | TICAM1 | Toll Like Receptor Adaptor Molecule 1 | 12hpi vs 24 hpi |
|  | TLR2-1 | the type1isoform of TLR2 | 12hpi vs 24 hpi |
|  | TLR3 | Toll-Like Receptor 3 | 12hpi vs 24 hpi |
|  | TRAF3 | TNF Receptor Associated Factor 3 | 12hpi vs 24 hpi |
|  | TRAF6 | TNF receptor-associated factor 6 | 12hpi vs 24 hpi |
| PI3K-Akt Signaling pathway | BCL2 | BCL2 Apoptosis Regulator | 0hpi vs 12 hpi |
|  | BCL2L1 | BCL2 Like 1 | 0hpi vs 12 hpi |
|  | CCNE1 | Cyclin E1 | 0hpi vs 12 hpi |
|  | COL6A2 | Collagen Type VI Alpha 2 Chain | 0hpi vs 12 hpi |
|  | COL6A3 | Collagen Type VI Alpha 3 Chain | 0hpi vs 12 hpi |
|  | CREB3L | cAMP-responsive element binding protein 3-like | 0hpi vs 12 hpi |
|  | EGFR | Epidermal Growth Factor Receptor | 0hpi vs 12 hpi |
|  | FGFR2 | Fibroblast Growth Factor Receptor 2 | 0hpi vs 12 hpi |
|  | FGFR3 | Fibroblast Growth Factor Receptor 3 | 0hpi vs 12 hpi |
|  | FOXO3 | Forkhead Box O3 | 0hpi vs 12 hpi |
|  | G6PC1 | Glucose-6-Phosphatase Catalytic Subunit 1 | 0hpi vs 12 hpi |
|  | IRS1 | insulin receptor substrates 1 | 0hpi vs 12 hpi |
|  | ITGA7 | Integrin Subunit Alpha 7 | 0hpi vs 12 hpi |
|  | ITGA9 | Integrin Subunit Alpha 9 | 0hpi vs 12 hpi |
|  | ITGB5 | Integrin Subunit Beta 5 | 0hpi vs 12 hpi |
|  | ITGB7 | Integrin Subunit Beta 7 | 0hpi vs 12 hpi |
|  | LAMA1 | Laminin Subunit Alpha 1 | 0hpi vs 12 hpi |
|  | NR4A1 | Nuclear Receptor Subfamily 4 Group A Member 1 | 0hpi vs 12 hpi |
|  | VEGFC | Vascular Endothelial Growth Factor C | 0hpi vs 12 hpi |
|  | VEGFD | Vascular Endothelial Growth Factor D | 0hpi vs 12 hpi |
|  | CDKN1A | Cyclin Dependent Kinase Inhibitor 1A | 12hpi vs 24 hpi |
|  | CREB3L1 | CAMP Responsive Element Binding Protein 3 Like 1 | 12hpi vs 24 hpi |
|  | CREB5 | CAMP Responsive Element Binding Protein 5 | 12hpi vs 24 hpi |
|  | EIF4E2 | Eukaryotic Translation Initiation Factor 4E Family Member 2 | 12hpi vs 24 hpi |
|  | TNFSF6 | TNF Superfamily Member 10 | 12hpi vs 24 hpi |
|  | IL6R | Interleukin 6 Receptor | 12hpi vs 24 hpi |
|  | ITGA8 | Integrin Subunit Alpha 8 | 12hpi vs 24 hpi |
|  | ITGB3 | Integrin Subunit Beta 3 | 12hpi vs 24 hpi |
|  | ITGB8 | Integrin Subunit Beta 8 | 12hpi vs 24 hpi |
|  | NFKB1 | Nuclear Factor Kappa B Subunit 1 | 12hpi vs 24 hpi |
|  | NR4A1 | Nuclear Receptor Subfamily 4 Group A Member 1 | 12hpi vs 24 hpi |
|  | PCK1 | Phosphoenolpyruvate Carboxykinase 1 | 12hpi vs 24 hpi |
|  | PDGFB | Platelet Derived Growth Factor Subunit B | 12hpi vs 24 hpi |
|  | PIK3AP1 | Phosphoinositide-3-Kinase Adaptor Protein 1 | 12hpi vs 24 hpi |
|  | PIK3CB | Phosphatidylinositol-4,5-Bisphosphate 3-Kinase Catalytic Subunit Beta | 12hpi vs 24 hpi |
|  | PIK3R5 | Phosphoinositide-3-Kinase Regulatory Subunit 5 | 12hpi vs 24 hpi |
|  | RELN | Reelin | 12hpi vs 24 hpi |
|  | THBS1 | Thrombospondin 1 | 12hpi vs 24 hpi |
|  | TLR2-1 | Toll-like receptor 2 | 12hpi vs 24 hpi |
| Endocytosis pathway | CALR3 | Calreticulin 3 | 0hpi vs 12 hpi |
|  | CAV1 | Caveolin 1 | 0hpi vs 12 hpi |
|  | DAB2 | DAB Adaptor Protein 2 | 0hpi vs 12 hpi |
|  | EGFR | Epidermal Growth Factor Receptor | 0hpi vs 12 hpi |
|  | FGFR2 | Fibroblast Growth Factor Receptor 2 | 0hpi vs 12 hpi |
|  | FGFR3 | Fibroblast Growth Factor Receptor 3 | 0hpi vs 12 hpi |
|  | IQSEC3 | IQ Motif and SEC7 Domain-containing Protein 3 | 0hpi vs 12 hpi |
|  | TGFB2 | Transforming Growth Factor Beta 2 | 0hpi vs 12 hpi |
|  | ARPC5L | Actin Related Protein 2/3 Complex Subunit 5 Like | 0hpi vs 12 hpi |
|  | ASAP1 | ArfGAP With SH3 Domain, Ankyrin Repeat And PH Domain 1 | 12hpi vs 24 hpi |
|  | CAV2 | Caveolin-2 | 12hpi vs 24 hpi |
|  | CXCR4 | C-X-C Motif Chemokine Receptor 4 | 12hpi vs 24 hpi |
|  | GRK7 | G Protein-Coupled Receptor Kinase 7 | 12hpi vs 24 hpi |
|  | HSPA8 | Heat Shock Protein Family A (Hsp70) Member 8 | 12hpi vs 24 hpi |
|  | IQSEC3 | IQ Motif and SEC7 Domain-containing Protein 3 | 12hpi vs 24 hpi |
|  | PIP5K1B | Phosphatidylinositol-4-Phosphate 5-Kinase Type 1 Beta | 12hpi vs 24 hpi |
|  | SH3GL2 | SH3 Domain Containing GRB2 Like 2, Endophilin A1 | 12hpi vs 24 hpi |
|  | SMURF1 | SMAD Specific E3 Ubiquitin Protein Ligase 1 | 12hpi vs 24 hpi |
|  | TRAF6 | TNF receptor-associated factor 6 | 12hpi vs 24 hpi |
| Cell cycle | CCNE1 | Cyclin E1 | 0hpi vs 12 hpi |
|  | CDKN2B | Cyclin Dependent Kinase Inhibitor 2B | 0hpi vs 12 hpi |
|  | MYC | MYC Proto-Oncogene, BHLH Transcription Factor | 0hpi vs 12 hpi |
|  | TGFB2 | Transforming Growth Factor Beta 2 | 0hpi vs 12 hpi |
|  | CCNB3 | Cyclin B3 | 12hpi vs 24 hpi |
|  | CDC20 | Cell Division Cycle 20 | 12hpi vs 24 hpi |
|  | CDKN1A | Cyclin Dependent Kinase Inhibitor 1A | 12hpi vs 24 hpi |
|  | GADD45B | Growth Arrest And DNA Damage Inducible Beta | 12hpi vs 24 hpi |
|  | GADD45G | Growth Arrest And DNA Damage Inducible Gamma | 12hpi vs 24 hpi |
|  | MYC | MYC Proto-Oncogene, BHLH Transcription Factor | 12hpi vs 24 hpi |
|  | PTTG2 | Pituitary Tumor-Transforming 2 | 12hpi vs 24 hpi |
| Tight juntion | BCL2 | BCL2 Apoptosis Regulator | 0hpi vs 12 hpi |
|  | CAV1 | Caveolin 1 | 0hpi vs 12 hpi |
|  | COL6A2 | Collagen Type VI Alpha 2 Chain | 0hpi vs 12 hpi |
|  | COL6A3 | Collagen Type VI Alpha 3 Chain | 0hpi vs 12 hpi |
|  | EGFR | Epidermal Growth Factor Receptor | 0hpi vs 12 hpi |
|  | ITGA7 | Integrin Subunit Alpha 7 | 0hpi vs 12 hpi |
|  | ITGA9 | Integrin Subunit Alpha 9 | 0hpi vs 12 hpi |
|  | ITGB5 | Integrin Subunit Beta 5 | 0hpi vs 12 hpi |
|  | ITGB7 | Integrin Subunit Beta 7 | 0hpi vs 12 hpi |
|  | LAMA1 | Laminin Subunit Alpha 1 | 0hpi vs 12 hpi |
|  | PIK3CB | Phosphatidylinositol-4,5-Bisphosphate 3-Kinase Catalytic Subunit Beta | 0hpi vs 12 hpi |
|  | PIK3R1 | Phosphoinositide-3-Kinase Regulatory Subunit 1 | 0hpi vs 12 hpi |
|  | PRKCB | Protein Kinase C Beta | 0hpi vs 12 hpi |
|  | VEGFC | Vascular Endothelial Growth Factor C | 0hpi vs 12 hpi |
|  | VEGFD | Vascular Endothelial Growth Factor D | 0hpi vs 12 hpi |
|  | CAV2 | Caveolin-2 | 12hpi vs 24 hpi |
|  | ITGA8 | Integrin Subunit Alpha 8 | 12hpi vs 24 hpi |
|  | ITGB3 | Integrin Subunit Beta 3 | 12hpi vs 24 hpi |
|  | ITGB8 | Integrin Subunit Beta 8 | 12hpi vs 24 hpi |
|  | JUN | Jun Proto-Oncogene, AP-1 Transcription Factor Subunit | 12hpi vs 24 hpi |
|  | LAMB3 | Laminin Subunit Beta 3 | 12hpi vs 24 hpi |
|  | MAPK10 | Mitogen-Activated Protein Kinase 10 | 12hpi vs 24 hpi |
|  | MYLK3 | Myosin Light Chain Kinase 3 | 12hpi vs 24 hpi |
|  | PDGFB | Platelet Derived Growth Factor Subunit B | 12hpi vs 24 hpi |
|  | PIK3CB | Phosphatidylinositol-4,5-Bisphosphate 3-Kinase Catalytic Subunit Beta | 12hpi vs 24 hpi |
| Thyroid hormone signaling pathway | BMP2 | Bone Morphogenetic Protein 2 | 0hpi vs 12 hpi |
|  | ATP1B1 | ATPase Na+/K+ Transporting Subunit Beta 1 | 0hpi vs 12 hpi |
|  | BMP4 | Bone Morphogenetic Protein 4 | 0hpi vs 12 hpi |
|  | DIO1 | Iodothyronine Deiodinase 1 | 0hpi vs 12 hpi |
|  | DIO2 | Iodothyronine Deiodinase 2 | 0hpi vs 12 hpi |
|  | ESR1 | Estrogen Receptor 1 | 0hpi vs 12 hpi |
|  | FOXO1 | Forkhead Box O1 | 0hpi vs 12 hpi |
|  | MYC | MYC Proto-Oncogene, BHLH Transcription Factor | 0hpi vs 12 hpi |
|  | PIK3CB | Phosphatidylinositol-4,5-Bisphosphate 3-Kinase Catalytic Subunit Beta | 0hpi vs 12 hpi |
|  | PIK3R1 | Phosphoinositide-3-Kinase Regulatory Subunit 1 | 0hpi vs 12 hpi |
|  | PLCB2 | Phospholipase C Beta 2 | 0hpi vs 12 hpi |
|  | PLCD1 | Phospholipase C Delta 1 | 0hpi vs 12 hpi |
|  | PLCE1 | Phospholipase C Epsilon 1 | 0hpi vs 12 hpi |
|  | PRKCB | Protein Kinase C Beta | 0hpi vs 12 hpi |
|  | RXRG | Retinoid X Receptor Gamma | 0hpi vs 12 hpi |
|  | SLC16A2 | Solute Carrier Family 16 Member 2 | 0hpi vs 12 hpi |
|  | SLCO1C1 | Solute Carrier Organic Anion Transporter Family Member 1C1 | 12hpi vs 24 hpi |
|  | DIO2 | Iodothyronine Deiodinase 2 | 12hpi vs 24 hpi |
|  | FOXO1 | Forkhead Box O1 | 12hpi vs 24 hpi |
|  | ITGB3 | Integrin Subunit Beta 3 | 12hpi vs 24 hpi |
|  | MYC | MYC Proto-Oncogene, BHLH Transcription Factor | 12hpi vs 24 hpi |
|  | NOTCH1 | Notch Receptor 1 | 12hpi vs 24 hpi |
|  | PIK3CB | Phosphatidylinositol-4,5-Bisphosphate 3-Kinase Catalytic Subunit Beta | 12hpi vs 24 hpi |
|  | SLC16A2 | Solute Carrier Family 16 Member 2 | 12hpi vs 24 hpi |
|  | WNT4 | Wnt Family Member 4 | 12hpi vs 24 hpi |
| Regulation of action cytoskeleton | ARHGEF4 | Rho Guanine Nucleotide Exchange Factor 4 | 0hpi vs 12 hpi |
|  | BAIAP2 | BAR/IMD Domain Containing Adaptor Protein 2 | 0hpi vs 12 hpi |
|  | CHRM5 | Cholinergic Receptor Muscarinic 5 | 0hpi vs 12 hpi |
|  | EGFR | Epidermal Growth Factor Receptor | 0hpi vs 12 hpi |
|  | FGFR2 | Fibroblast Growth Factor Receptor 2 | 0hpi vs 12 hpi |
|  | FGFR3 | Fibroblast Growth Factor Receptor 3 | 0hpi vs 12 hpi |
|  | ITGA7 | Integrin Subunit Alpha 7 | 0hpi vs 12 hpi |
|  | ITGA9 | Integrin Subunit Alpha 9 | 0hpi vs 12 hpi |
|  | ITGB5 | Integrin Subunit Beta 5 | 0hpi vs 12 hpi |
|  | ITGB7 | Integrin Subunit Beta 7 | 0hpi vs 12 hpi |
|  | MYLK | Myosin Light Chain Kinase | 0hpi vs 12 hpi |
|  | PDGFA | Platelet Derived Growth Factor Subunit A | 0hpi vs 12 hpi |
|  | PIK3CB | Phosphatidylinositol-4,5-Bisphosphate 3-Kinase Catalytic Subunit Beta | 0hpi vs 12 hpi |
|  | PIK3R1 | Phosphoinositide-3-Kinase Regulatory Subunit 1 | 0hpi vs 12 hpi |
|  | ABHD4 | Abhydrolase Domain Containing 4, N-Acyl Phospholipase B | 12hpi vs 24 hpi |
|  | ADCY3 | Adenylate Cyclase 3 | 12hpi vs 24 hpi |
|  | PIK3CB | Phosphatidylinositol-4,5-Bisphosphate 3-Kinase Catalytic Subunit Beta | 12hpi vs 24 hpi |
|  | PTGS2 | Prostaglandin-Endoperoxide Synthase 2 | 12hpi vs 24 hpi |
