# Supplementary material for: Transcriptome Analysis of Genes Responding to Infection of Leghorn Male Hepatocellular Cells With Fowl Adenovirus Serotype 4
Source: Front Vet Sci. 2022 Jun 14;9:871038. doi: 10.3389/fvets.2022.871038 (PMC9237548; doi:10.3389/fvets.2022.871038)
Supplement: Supplementary file 13 [file Table_7.DOCX]

Differentially expressed gene (DEGs) involved in host immunosuppressive to FAdV-4 in LMH cells at 12, 24,36,48,60h

| Biological process | Gene symbol | Gene description | Source of DEGs |
| --- | --- | --- | --- |
| Apoptosis - fly | DUSP10 | Dual Specificity Phosphatase 10 | 0hpi vs 12 hpi |
|  | EGFR | Epidermal Growth Factor Receptor | 0hpi vs 12 hpi |
|  | EIF2AK3 | Eukaryotic Translation Initiation Factor 2 Alpha Kinase 3 | 0hpi vs 12 hpi |
|  | BCL2 | BCL2 Apoptosis Regulator | 12 hpi vs 24hpi |
|  | BCL2L1 | BCL2 Like 1 | 12 hpi vs 24hpi |
|  | BIRC7 | Baculoviral IAP Repeat Containing 7 | 12 hpi vs 24hpi |
|  | CASP3 | Caspase 3 | 12 hpi vs 24hpi |
|  | TNFRSF1A | TNF Receptor Superfamily Member 1A | 12 hpi vs 24hpi |
|  | JUN | Jun Proto-Oncogene, AP-1 Transcription Factor Subunit | 12 hpi vs 24hpi |
|  | KRAS | KRAS Proto-Oncogene, GTPase | 24hpi vs 36hpi |
|  | MAP3K5 | Mitogen-Activated Protein Kinase Kinase Kinase 5 | 24hpi vs 36hpi |
|  | MAPK10 | Mitogen-Activated Protein Kinase 10 | 24hpi vs 36hpi |
|  | DUSP10 | Dual Specificity Phosphatase 10 | 36hpi vs 48hpi |
|  | JUN | Jun Proto-Oncogene, AP-1 Transcription Factor Subunit | 36hpi vs 48hpi |
|  | KRAS | KRAS Proto-Oncogene, GTPase | 36hpi vs 48hpi |
|  | MAP3K5 | Mitogen-Activated Protein Kinase Kinase Kinase 5 | 36hpi vs 48hpi |
|  | MAPK10 | Mitogen-Activated Protein Kinase 10 | 36hpi vs 48hpi |
|  | ATF4 | Activating Transcription Factor 4 | 48hpi vs 60hpi |
|  | ATM | ATM Serine/Threonine Kinase | 48hpi vs 60hpi |
|  | DUSP10 | Dual Specificity Phosphatase 10 | 48hpi vs 60hpi |
|  | JUN | Jun Proto-Oncogene, AP-1 Transcription Factor Subunit | 48hpi vs 60hpi |
|  | TRAF4 | TNF Receptor Associated Factor 4 | 48hpi vs 60hpi |
|  | MAP3K5 | Mitogen-Activated Protein Kinase Kinase Kinase 5 | 48hpi vs 60hpi |
|  | MAPK10 | Mitogen-Activated Protein Kinase 10 | 48hpi vs 60hpi |
|  | EGFR | Epidermal Growth Factor Receptor | 48hpi vs 60hpi |
| Apoptosis-multiple species | BCL2 | BCL2 Apoptosis Regulator | 0hpi vs 12 hpi |
|  | BCL2L1 | BCL2 Like 1 | 0hpi vs 12 hpi |
|  | BIRC7 | Baculoviral IAP Repeat Containing 7 | 0hpi vs 12 hpi |
|  | CASP3 | Caspase 3 | 0hpi vs 12 hpi |
|  | TNFRSF1A | TNF Receptor Superfamily Member 1A | 0hpi vs 12 hpi |
|  | BCL2L1 | BCL2 Like 1 | 12hpi vs 24 hpi |
|  | FADD | Fas Associated Via Death Domain | 12hpi vs 24 hpi |
|  | MAPK10 | Mitogen-Activated Protein Kinase 10 | 12hpi vs 24 hpi |
|  | BCL2L1 | BCL2 Like 1 | 24hpi vs 36 hpi |
|  | MAPK10 | Mitogen-Activated Protein Kinase 10 | 24hpi vs 36 hpi |
|  | MAPK10 | Mitogen-Activated Protein Kinase 10 | 36hpi vs 48 hpi |
|  | CASP9 | Caspase 9 | 48hpi vs 60 hpi |
|  | MAPK10 | Mitogen-Activated Protein Kinase 10 | 48hpi vs 60 hpi |
|  | TNFRSF1A | TNF Receptor Superfamily Member 1A | 48hpi vs 60 hpi |
| Inflammatory mediator regulation of TRP channels | F2RL1 | F2R Like Trypsin Receptor 1 | 0hpi vs 12 hpi |
|  | IL1R1 | Interleukin 1 Receptor Type 1 | 0hpi vs 12 hpi |
|  | PIK3CB | Phosphatidylinositol-4,5-Bisphosphate 3-Kinase Catalytic Subunit Beta | 0hpi vs 12 hpi |
|  | PIK3R1 | Phosphoinositide-3-Kinase Regulatory Subunit 1 | 0hpi vs 12 hpi |
|  | PLCB2 | Phospholipase C Beta 2 | 0hpi vs 12 hpi |
|  | PRKCB | Protein Kinase C Beta | 0hpi vs 12 hpi |
|  | TRPV2 | Transient Receptor Potential Cation Channel Subfamily V Member 2 | 0hpi vs 12 hpi |
|  | ADCY3 | Adenylate Cyclase 3 | 12hpi vs 24 hpi |
|  | CYP2C18 | Cytochrome P450 Family 2 Subfamily C Member 18 | 12hpi vs 24 hpi |
|  | F2RL1 | F2R Like Trypsin Receptor 1 | 12hpi vs 24 hpi |
|  | IL1R1 | Interleukin 1 Receptor Type 1 | 12hpi vs 24 hpi |
|  | MAPK10 | Mitogen-Activated Protein Kinase 10 | 12hpi vs 24 hpi |
|  | PIK3CB | Phosphatidylinositol-4,5-Bisphosphate 3-Kinase Catalytic Subunit Beta | 12hpi vs 24 hpi |
|  | PRKCD | Protein Kinase C Delta | 12hpi vs 24 hpi |
|  | TRPV2 | Transient Receptor Potential Cation Channel Subfamily V Member 2 | 12hpi vs 24 hpi |
|  | ADCY3 | Adenylate Cyclase 3 | 24hpi vs 36 hpi |
|  | BDKRB1 | Bradykinin Receptor B1 | 24hpi vs 36 hpi |
|  | BDKRB2 | Bradykinin Receptor B2 | 24hpi vs 36 hpi |
|  | CYP2C18 | Cytochrome P450 Family 2 Subfamily C Member 18 | 24hpi vs 36 hpi |
|  | F2RL1 | F2R Like Trypsin Receptor 1 | 24hpi vs 36 hpi |
|  | IL1R1 | Interleukin 1 Receptor Type 1 | 24hpi vs 36 hpi |
|  | MAPK10 | Mitogen-Activated Protein Kinase 10 | 24hpi vs 36 hpi |
|  | PLA2G4F | Phospholipase A2 Group IVF | 24hpi vs 36 hpi |
|  | PRKCD | Protein Kinase C Delta | 24hpi vs 36 hpi |
|  | TRPA1 | Transient Receptor Potential Cation Channel Subfamily A Member 1 | 24hpi vs 36 hpi |
|  | ADCY2 | Adenylate Cyclase 2 | 36hpi vs 48 hpi |
|  | ADCY8 | Adenylate Cyclase 8 | 36hpi vs 48 hpi |
|  | BDKRB2 | Bradykinin Receptor B2 | 36hpi vs 48 hpi |
|  | CYP2C18 | Cytochrome P450 Family 2 Subfamily C Member 18 | 36hpi vs 48 hpi |
|  | F2RL1 | F2R Like Trypsin Receptor 1 | 36hpi vs 48 hpi |
|  | HTR2A | 5-Hydroxytryptamine Receptor 2A | 36hpi vs 48 hpi |
|  | HTR2C | 5-Hydroxytryptamine Receptor 2C | 36hpi vs 48 hpi |
|  | MAPK10 | Mitogen-Activated Protein Kinase 10 | 36hpi vs 48 hpi |
|  | NGF | Nerve Growth Factor | 36hpi vs 48 hpi |
|  | PLA2G4F | Phospholipase A2 Group IVF | 36hpi vs 48 hpi |
|  | PRKCD | Protein Kinase C Delta | 36hpi vs 48 hpi |
|  | SRC | SRC Proto-Oncogene, Non-Receptor Tyrosine Kinase | 36hpi vs 48 hpi |
|  | TRPA1 | Transient Receptor Potential Cation Channel Subfamily A Member 1 | 36hpi vs 48 hpi |
|  | TRPV1 | Transient Receptor Potential Cation Channel Subfamily V Member 1 | 36hpi vs 48 hpi |
|  | TRPV2 | Transient Receptor Potential Cation Channel Subfamily V Member 2 | 36hpi vs 48 hpi |
|  | ADCY7 | Adenylate Cyclase 7 | 48hpi vs 60 hpi |
|  | ADCY8 | Adenylate Cyclase 8 | 48hpi vs 60 hpi |
|  | BDKRB2 | Bradykinin Receptor B2 | 48hpi vs 60 hpi |
|  | HTR2C | 5-Hydroxytryptamine Receptor 2C | 48hpi vs 60 hpi |
|  | IL1R1 | Interleukin 1 Receptor Type 1 | 48hpi vs 60 hpi |
|  | MAPK10 | Mitogen-Activated Protein Kinase 10 | 48hpi vs 60 hpi |
|  | MAPK12 | Mitogen-Activated Protein Kinase 12 | 48hpi vs 60 hpi |
|  | NGF | Nerve Growth Factor | 48hpi vs 60 hpi |
|  | PIK3CB | Phosphatidylinositol-4,5-Bisphosphate 3-Kinase Catalytic Subunit Beta | 48hpi vs 60 hpi |
|  | PIK3CD | Phosphatidylinositol-4,5-Bisphosphate 3-Kinase Catalytic Subunit Delta | 48hpi vs 60 hpi |
|  | PIK3R1 | Phosphoinositide-3-Kinase Regulatory Subunit 1 | 48hpi vs 60 hpi |
|  | PLA2G4F | Phospholipase A2 Group IVF | 48hpi vs 60 hpi |
|  | PLCB1 | Phospholipase C Beta 1 | 48hpi vs 60 hpi |
|  | PRKCD | Protein Kinase C Delta | 48hpi vs 60 hpi |
|  | PTGER4 | Prostaglandin E Receptor 4 | 48hpi vs 60 hpi |
|  | TRPV1 | Transient Receptor Potential Cation Channel Subfamily V Member 1 | 48hpi vs 60 hpi |
|  | TRPV2 | Transient Receptor Potential Cation Channel Subfamily V Member 2 | 48hpi vs 60 hpi |
| Toll-like receptor signaling pathway | CD40 | Clusters of differentiation 40 | 0hpi vs 12 hpi |
|  | CD80 | Clusters of differentiation 80 | 0hpi vs 12 hpi |
|  | FOS | Fos Proto-Oncogene, AP-1 Transcription Factor Subunit | 0hpi vs 12 hpi |
|  | IRF7 | Interferon regulatory factor 7 | 0hpi vs 12 hpi |
|  | PIK3R1 | Phosphoinositide-3-Kinase Regulatory Subunit 1 | 0hpi vs 12 hpi |
|  |  |  |  |
|  | TLR5 | Toll-Like Receptor 5 | 0hpi vs 12 hpi |
|  | FADD | Fas Associated Via Death Domain | 12hpi vs 24 hpi |
|  | FOS | Fos Proto-Oncogene, AP-1 Transcription Factor Subunit | 12hpi vs 24 hpi |
|  | IL-12B | Interleukin 12B | 12hpi vs 24 hpi |
|  | IL8 | Interleukin-8 | 12hpi vs 24 hpi |
|  | IRF7 | Interferon regulatory factor 7 | 12hpi vs 24 hpi |
|  | JUN | Jun Proto-Oncogene, AP-1 Transcription Factor Subunit | 12hpi vs 24 hpi |
|  | MAP3K8 | Mitogen-Activated Protein Kinase Kinase Kinase 8 | 12hpi vs 24 hpi |
|  | MAPK10 | Mitogen-Activated Protein Kinase 10 | 12hpi vs 24 hpi |
|  | NFKB1 | Nuclear Factor Kappa B Subunit 1 | 12hpi vs 24 hpi |
|  | PIK3CB | Phosphatidylinositol-4,5-Bisphosphate 3-Kinase Catalytic Subunit Beta | 12hpi vs 24 hpi |
|  | SPP1 | Secreted Phosphoprotein 1 | 12hpi vs 24 hpi |
|  | TICAM1 | Toll Like Receptor Adaptor Molecule 1 | 12hpi vs 24 hpi |
|  | TLR3 | Toll-Like Receptor 3 | 12hpi vs 24 hpi |
|  | TLR5 | Toll-Like Receptor 5 | 12hpi vs 24 hpi |
|  | TRAF3 | TNF Receptor Associated Factor 3 | 12hpi vs 24 hpi |
|  | TRAF6 | TNF Receptor Associated Factor 6 | 12hpi vs 24 hpi |
|  | FOS | Fos Proto-Oncogene, AP-1 Transcription Factor Subunit | 24hpi vs 36hpi |
|  | IL-12B | Interleukin 12B | 24hpi vs 36hpi |
|  | IL8 | Interleukin-8 | 24hpi vs 36hpi |
|  | IRF7 | Interferon regulatory factor 7 | 24hpi vs 36hpi |
|  | JUN | Jun Proto-Oncogene, AP-1 Transcription Factor Subunit | 24hpi vs 36hpi |
|  | MAP3K8 | Mitogen-Activated Protein Kinase Kinase Kinase 8 | 24hpi vs 36hpi |
|  | MAPK10 | Mitogen-Activated Protein Kinase 10 | 24hpi vs 36hpi |
|  | NFKB1 | Nuclear Factor Kappa B Subunit 1 | 24hpi vs 36hpi |
|  | NFKBIA | NFKB Inhibitor Alpha | 24hpi vs 36hpi |
|  | TLR5 | Toll-Like Receptor 5 | 24hpi vs 36hpi |
|  | TRAF3 | TNF Receptor Associated Factor 3 | 24hpi vs 36hpi |
|  | CD80 | Clusters of differentiation 80 | 36hpi vs 48 hpi |
|  | FOS | Fos Proto-Oncogene, AP-1 Transcription Factor Subunit | 36hpi vs 48 hpi |
|  | IL-12B | Interleukin 12B | 36hpi vs 48 hpi |
|  | IL8 | Interleukin-8 | 36hpi vs 48 hpi |
|  |  |  |  |
|  | IRF7 | Interferon regulatory factor 7 | 36hpi vs 48 hpi |
|  | JUN | Jun Proto-Oncogene, AP-1 Transcription Factor Subunit | 36hpi vs 48 hpi |
|  | MAP3K8 | Mitogen-Activated Protein Kinase Kinase Kinase 8 | 36hpi vs 48 hpi |
|  | MAPK10 | Mitogen-Activated Protein Kinase 10 | 36hpi vs 48 hpi |
|  | NFKB1 | Nuclear Factor Kappa B Subunit 1 | 36hpi vs 48 hpi |
|  | TLR5 | Toll-Like Receptor 5 | 36hpi vs 48 hpi |
|  | TRAF3 | TNF Receptor Associated Factor 3 | 36hpi vs 48 hpi |
|  | FOS | Fos Proto-Oncogene, AP-1 Transcription Factor Subunit | 48hpi vs 60 hpi |
|  | IKBKE | Inhibitor Of Nuclear Factor Kappa B Kinase Subunit Epsilon | 48hpi vs 60 hpi |
|  | IL-12B | Interleukin 12B | 48hpi vs 60 hpi |
|  | IL8 | Interleukin-8 | 48hpi vs 60 hpi |
|  | IRF7 | Interferon regulatory factor 7 | 48hpi vs 60 hpi |
|  | JUN | Jun Proto-Oncogene, AP-1 Transcription Factor Subunit | 48hpi vs 60 hpi |
|  | MAP3K8 | Mitogen-Activated Protein Kinase Kinase Kinase 8 | 48hpi vs 60 hpi |
|  | MAPK10 | Mitogen-Activated Protein Kinase 10 | 48hpi vs 60 hpi |
|  | MAPK12 | Mitogen-Activated Protein Kinase 12 | 48hpi vs 60 hpi |
|  | NFKB1 | Nuclear Factor Kappa B Subunit 1 | 48hpi vs 60 hpi |
|  | NFKBIA | NFKB Inhibitor Alpha | 48hpi vs 60 hpi |
|  | PIK3CB | Phosphatidylinositol-4,5-Bisphosphate 3-Kinase Catalytic Subunit Beta | 48hpi vs 60 hpi |
|  | PIK3CD | Phosphatidylinositol-4,5-Bisphosphate 3-Kinase Catalytic Subunit Delta | 48hpi vs 60 hpi |
|  | PIK3R1 | Phosphoinositide-3-Kinase Regulatory Subunit 1 | 48hpi vs 60 hpi |
|  | TBK1 | TANK Binding Kinase 1 | 48hpi vs 60 hpi |
|  | TICAM1 | Toll Like Receptor Adaptor Molecule 1 | 48hpi vs 60 hpi |
|  | TRAF3 | TNF Receptor Associated Factor 3 | 48hpi vs 60 hpi |
| Jak-STAT signaling pathway | BCL2 | BCL2 Apoptosis Regulator | 0hpi vs 12 hpi |
|  | BCL2L1 | BCL2 Like 1 | 0hpi vs 12 hpi |
|  | CNTFR | Ciliary Neurotrophic Factor Receptor | 0hpi vs 12 hpi |
|  | IL13RA1 | Interleukin 13 Receptor Subunit Alpha 1 | 0hpi vs 12 hpi |
|  | IL15 | Interleukin 15 | 0hpi vs 12 hpi |
|  | IL20RA | Interleukin 20 Receptor Subunit Alpha | 0hpi vs 12 hpi |
|  | IL21R | Interleukin 21 Receptor | 0hpi vs 12 hpi |
|  | IL22RA1 | Interleukin 22 Receptor Subunit Alpha 1 | 0hpi vs 12 hpi |
|  | IL22RA2 | Interleukin 22 Receptor Subunit Alpha 2 | 0hpi vs 12 hpi |
|  | LIFR | LIF Receptor Subunit Alpha | 0hpi vs 12 hpi |
|  | MYC | MYC Proto-Oncogene, BHLH Transcription Facto | 0hpi vs 12 hpi |
|  | PIK3CB | Phosphatidylinositol-4,5-Bisphosphate 3-Kinase Catalytic Subunit Beta | 0hpi vs 12 hpi |
|  | PIK3R1 | Phosphoinositide-3-Kinase Regulatory Subunit 1 | 0hpi vs 12 hpi |
|  | PIM1 | Pim-1 Proto-Oncogene, Serine/Threonine Kinase | 0hpi vs 12 hpi |
|  | SOCS2 | Suppressor Of Cytokine Signaling 2 | 0hpi vs 12 hpi |
|  | SOCS3 | Suppressor Of Cytokine Signaling 3 | 0hpi vs 12 hpi |
|  | BCL2L1 | BCL2 Like 1 | 12hpi vs 24 hpi |
|  | CDKN1A | Cyclin Dependent Kinase Inhibitor 1A | 12hpi vs 24 hpi |
|  | IL-12B | Interleukin 12B | 12hpi vs 24 hpi |
|  | IL13RA2 | Interleukin 13 Receptor Subunit Alpha 2 | 12hpi vs 24 hpi |
|  | IL17D | Interleukin 17D | 12hpi vs 24 hpi |
|  | IL22RA1 | Interleukin 22 Receptor Subunit Alpha 1 | 12hpi vs 24 hpi |
|  | IL6R | Interleukin 6 Receptor | 12hpi vs 24 hpi |
|  | MYC | MYC Proto-Oncogene, BHLH Transcription Facto | 12hpi vs 24 hpi |
|  | PIK3CB | Phosphatidylinositol-4,5-Bisphosphate 3-Kinase Catalytic Subunit Beta | 12hpi vs 24 hpi |
|  | PIM1 | Pim-1 Proto-Oncogene, Serine/Threonine Kinase | 12hpi vs 24 hpi |
|  | SOCS1 | Suppressor Of Cytokine Signaling 1 | 12hpi vs 24 hpi |
|  | SOCS3 | Suppressor Of Cytokine Signaling 3 | 12hpi vs 24 hpi |
|  | SOCS6 | Suppressor Of Cytokine Signaling 6 | 12hpi vs 24 hpi |
|  | AOX1 | Aldehyde Oxidase 1 | 24hpi vs 36hpi |
|  | BCL2L1 | BCL2 Like 1 | 24hpi vs 36hpi |
|  | CISH | Cytokine Inducible SH2 Containing Protein | 24hpi vs 36hpi |
|  | IL-12B | Interleukin 12B | 24hpi vs 36hpi |
|  |  |  |  |
|  | IL11RA | Interleukin 11 Receptor Subunit Alpha | 24hpi vs 36hpi |
|  | IL13RA2 | Interleukin 13 Receptor Subunit Alpha 2 | 24hpi vs 36hpi |
|  | IL20RA | Interleukin 20 Receptor Subunit Alpha | 24hpi vs 36hpi |
|  | IL21R | Interleukin 21 Receptor | 24hpi vs 36hpi |
|  | IL2RB | Interleukin 2 Receptor Subunit Beta | 24hpi vs 36hpi |
|  | MYC | MYC Proto-Oncogene, BHLH Transcription Facto | 24hpi vs 36hpi |
|  | SOCS1 | Suppressor Of Cytokine Signaling 1 | 24hpi vs 36hpi |
|  | SOCS3 | Suppressor Of Cytokine Signaling 3 | 24hpi vs 36hpi |
|  | STAT5B | Signal Transducer And Activator Of Transcription 5B | 24hpi vs 36hpi |
|  | CDKN1A | Cyclin Dependent Kinase Inhibitor 1A | 36hpi vs 48 hpi |
|  | CISH | Cytokine Inducible SH2 Containing Protein | 36hpi vs 48 hpi |
|  | IL-12B | Interleukin 12B | 36hpi vs 48 hpi |
|  | IL11RA | Interleukin 11 Receptor Subunit Alpha | 36hpi vs 48 hpi |
|  | IL13RA2 | Interleukin 13 Receptor Subunit Alpha 2 | 36hpi vs 48 hpi |
|  | IL20RA | Interleukin 20 Receptor Subunit Alpha | 36hpi vs 48 hpi |
|  | IL21R | Interleukin 21 Receptor | 36hpi vs 48 hpi |
|  | IL2RA | Interleukin 2 Receptor Subunit Alpha | 36hpi vs 48 hpi |
|  | MYC | MYC Proto-Oncogene, BHLH Transcription Facto | 36hpi vs 48 hpi |
|  | PIK3CB | Phosphatidylinositol-4,5-Bisphosphate 3-Kinase Catalytic Subunit Beta | 36hpi vs 48 hpi |
|  | PIK3CD | Phosphatidylinositol-4,5-Bisphosphate 3-Kinase Catalytic Subunit Delta | 36hpi vs 48 hpi |
|  | PIM1 | Pim-1 Proto-Oncogene, Serine/Threonine Kinase | 36hpi vs 48 hpi |
|  | SOCS1 | Suppressor Of Cytokine Signaling 1 | 36hpi vs 48 hpi |
|  | SOCS3 | Suppressor Of Cytokine Signaling 3 | 36hpi vs 48 hpi |
|  | STAT5B | Signal Transducer And Activator Of Transcription 5B | 36hpi vs 48 hpi |
|  | IL-12B | Interleukin 12B | 48hpi vs 60 hpi |
|  | IL13RA1 | Interleukin 13 Receptor Subunit Alpha 1 | 48hpi vs 60 hpi |
|  | IL13RA2 | Interleukin 13 Receptor Subunit Alpha 2 | 48hpi vs 60 hpi |
|  | IL20RA | Interleukin 20 Receptor Subunit Alpha | 48hpi vs 60 hpi |
|  | IL21R | Interleukin 21 Receptor | 48hpi vs 60 hpi |
|  | IL22RA1 | Interleukin 22 Receptor Subunit Alpha 1 | 48hpi vs 60 hpi |
|  | IL2RA | Interleukin 2 Receptor Subunit Alpha | 48hpi vs 60 hpi |
|  | IL2RB | Interleukin 2 Receptor Subunit Beta | 48hpi vs 60 hpi |
|  | IL5RA | Interleukin 5 Receptor Subunit Alpha | 48hpi vs 60 hpi |
|  | MYC | MYC Proto-Oncogene, BHLH Transcription Facto | 48hpi vs 60 hpi |
|  | PIK3CB | Phosphatidylinositol-4,5-Bisphosphate 3-Kinase Catalytic Subunit Beta | 48hpi vs 60 hpi |
|  | PIK3CD | Phosphatidylinositol-4,5-Bisphosphate 3-Kinase Catalytic Subunit Delta | 48hpi vs 60 hpi |
|  | PIK3R1 | Phosphoinositide-3-Kinase Regulatory Subunit 1 | 48hpi vs 60 hpi |
|  | SOCS1 | Suppressor Of Cytokine Signaling 1 | 48hpi vs 60 hpi |
|  | SOCS3 | Suppressor Of Cytokine Signaling 3 | 48hpi vs 60 hpi |
| NF-kappa B signaling pathway | BCL2 | BCL2 Apoptosis Regulator | 0hpi vs 12 hpi |
|  | BCL2L1 | BCL2 Like 1 | 0hpi vs 12 hpi |
|  | BTK | Bruton Tyrosine Kinase | 0hpi vs 12 hpi |
|  | CD40 | Clusters of differentiation 40 | 0hpi vs 12 hpi |
|  | IL1R1 | Interleukin 1 Receptor Type 1 | 0hpi vs 12 hpi |
|  | TNFRSF1A | TNF Receptor Superfamily Member 1A | 0hpi vs 12 hpi |
|  | BCL2L1 | BCL2 Like 1 | 12hpi vs 24 hpi |
|  | CXCL12 | C-X-C Motif Chemokine Ligand 12 | 12hpi vs 24 hpi |
|  | IL1R1 | Interleukin 1 Receptor Type 1 | 12hpi vs 24 hpi |
|  | IL8 | Interleukin-8 | 12hpi vs 24 hpi |
|  | LCK | LCK Proto-Oncogene, Src Family Tyrosine Kinase | 12hpi vs 24 hpi |
|  | NFKB1 | Nuclear Factor Kappa B Subunit 1 | 12hpi vs 24 hpi |
|  | NFKBIA | NFKB Inhibitor Alpha | 12hpi vs 24 hpi |
|  | PTGS2 | Prostaglandin-Endoperoxide Synthase 2 | 12hpi vs 24 hpi |
|  | TNFAIP3 | TNF Alpha Induced Protein 3 | 12hpi vs 24 hpi |
|  | TNFSF11 | TNF Superfamily Member 11 | 12hpi vs 24 hpi |
|  | TNFSF13B | TNF Superfamily Member 13b | 12hpi vs 24 hpi |
|  | TRAF3 | TNF Receptor Associated Factor 3 | 12hpi vs 24 hpi |
|  | TRAF6 | TNF Receptor Associated Factor 6 | 12hpi vs 24 hpi |
|  | BCL2L1 | BCL2 Like 1 | 24hpi vs 36hpi |
|  | CXCL12 | C-X-C Motif Chemokine Ligand 12 | 24hpi vs 36hpi |
|  | NFKB1 | Nuclear Factor Kappa B Subunit 1 | 24hpi vs 36hpi |
|  | NFKBIA | NFKB Inhibitor Alpha | 24hpi vs 36hpi |
|  | PTGS2 | Prostaglandin-Endoperoxide Synthase 2 | 24hpi vs 36hpi |
|  | TNFAIP3 | TNF Alpha Induced Protein 3 | 24hpi vs 36hpi |
|  | TNFSF11 | TNF Superfamily Member 11 | 24hpi vs 36hpi |
|  | TRAF1 | TNF Receptor Associated Factor 1 | 24hpi vs 36hpi |
|  | TRAF3 | TNF Receptor Associated Factor 3 | 24hpi vs 36hpi |
|  | CARD11 | Caspase Recruitment Domain Family Member 11 | 36hpi vs 48 hpi |
|  | CXCL12 | C-X-C Motif Chemokine Ligand 12 | 36hpi vs 48 hpi |
|  | IL8 | Interleukin-8 | 36hpi vs 48 hpi |
|  | NFKB1 | Nuclear Factor Kappa B Subunit 1 | 36hpi vs 48 hpi |
|  | NFKB2 | Nuclear Factor Kappa B Subunit 2 | 36hpi vs 48 hpi |
|  | NFKBIA | NFKB Inhibitor Alpha | 36hpi vs 48 hpi |
|  | PTGS2 | Prostaglandin-Endoperoxide Synthase 2 | 36hpi vs 48 hpi |
|  | TNFAIP3 | TNF Alpha Induced Protein 3 | 36hpi vs 48 hpi |
|  | TNFSF11 | TNF Superfamily Member 11 | 36hpi vs 48 hpi |
|  | TRAF1， | TNF Receptor Associated Factor 1 | 36hpi vs 48 hpi |
|  | TRAF3 | TNF Receptor Associated Factor 3 | 36hpi vs 48 hpi |
|  | BCL2A1 | BCL2 Related Protein A1 | 48hpi vs 60 hpi |
|  | CXCL12 | C-X-C Motif Chemokine Ligand 12 | 48hpi vs 60 hpi |
|  | IL1R1 | Interleukin 1 Receptor Type 1 | 48hpi vs 60 hpi |
|  | IL8 | Interleukin-8 | 48hpi vs 60 hpi |
|  | NFKB1 | Nuclear Factor Kappa B Subunit 1 | 48hpi vs 60 hpi |
|  | NFKBIA | NFKB Inhibitor Alpha | 48hpi vs 60 hpi |
|  | PTGS2 | Prostaglandin-Endoperoxide Synthase 2 | 48hpi vs 60 hpi |
|  | TNFAIP3 | TNF Alpha Induced Protein 3 | 48hpi vs 60 hpi |
|  | TNFRSF1A | TNF Receptor Superfamily Member 1A | 48hpi vs 60 hpi |
|  | TNFSF11 | TNF Superfamily Member 11 | 48hpi vs 60 hpi |
|  | TNFSF13B | TNF Superfamily Member 13b | 48hpi vs 60 hpi |
| B cell receptor signaling pathway | CD81 | CD81 Molecule | 0hpi vs 12 hpi |
|  | FOS | Fos Proto-Oncogene, AP-1 Transcription Factor Subunit | 0hpi vs 12 hpi |
|  | NFKBIE | NFKB Inhibitor Epsilon | 0hpi vs 12 hpi |
|  | PIK3CB | Phosphatidylinositol-4,5-Bisphosphate 3-Kinase Catalytic Subunit Beta | 0hpi vs 12 hpi |
|  | PIK3R1 | Phosphoinositide-3-Kinase Regulatory Subunit 1 | 0hpi vs 12 hpi |
|  | FOS | Fos Proto-Oncogene, AP-1 Transcription Factor Subunit | 12hpi vs 24 hpi |
|  | JUN | Jun Proto-Oncogene, AP-1 Transcription Factor Subunit | 12hpi vs 24 hpi |
|  | NFKB1 | Nuclear Factor Kappa B Subunit 1 | 12hpi vs 24 hpi |
|  | NFKBIA | NFKB Inhibitor Alpha | 12hpi vs 24 hpi |
|  | NFKBIE | NFKB Inhibitor Epsilon | 12hpi vs 24 hpi |
|  | PIK3AP1 | Phosphoinositide-3-Kinase Adaptor Protein 1 | 12hpi vs 24 hpi |
|  | PIK3CB | Phosphatidylinositol-4,5-Bisphosphate 3-Kinase Catalytic Subunit Beta | 12hpi vs 24 hpi |
|  | FOS | Fos Proto-Oncogene, AP-1 Transcription Factor Subunit | 24hpi vs 36hpi |
|  | JUN | Jun Proto-Oncogene, AP-1 Transcription Factor Subunit | 24hpi vs 36hpi |
|  | NFKB1 | Nuclear Factor Kappa B Subunit 1 | 24hpi vs 36hpi |
|  | NFKBIA | NFKB Inhibitor Alpha | 24hpi vs 36hpi |
|  | NFKBIE | NFKB Inhibitor Epsilon | 24hpi vs 36hpi |
|  | JUN | Jun Proto-Oncogene, AP-1 Transcription Factor Subunit | 36hpi vs 48 hpi |
|  | KRAS | KRAS Proto-Oncogene, GTPase | 36hpi vs 48 hpi |
|  | NFATC2 | Nuclear Factor Of Activated T Cells 2 | 36hpi vs 48 hpi |
|  | NFKB1 | Nuclear Factor Kappa B Subunit 1 | 36hpi vs 48 hpi |
|  | NFKBIA | NFKB Inhibitor Alpha | 36hpi vs 48 hpi |
|  | NFKBIE | NFKB Inhibitor Epsilon | 36hpi vs 48 hpi |
|  | PIK3AP1 | Phosphoinositide-3-Kinase Adaptor Protein 1 | 36hpi vs 48 hpi |
|  | PIK3CB | Phosphatidylinositol-4,5-Bisphosphate 3-Kinase Catalytic Subunit Beta | 36hpi vs 48 hpi |
|  | PIK3CD | Phosphatidylinositol-4,5-Bisphosphate 3-Kinase Catalytic Subunit Delta | 36hpi vs 48 hpi |
|  | JUN | Jun Proto-Oncogene, AP-1 Transcription Factor Subunit | 36hpi vs 48 hpi |
|  | NFKB1 | Nuclear Factor Kappa B Subunit 1 | 36hpi vs 48 hpi |
|  | NFKBIA | NFKB Inhibitor Alpha | 36hpi vs 48 hpi |
|  | NFKBIE | NFKB Inhibitor Epsilon | 36hpi vs 48 hpi |
|  | PIK3CB | Phosphatidylinositol-4,5-Bisphosphate 3-Kinase Catalytic Subunit Beta | 36hpi vs 48 hpi |
|  | PIK3CD | Phosphatidylinositol-4,5-Bisphosphate 3-Kinase Catalytic Subunit Delta | 36hpi vs 48 hpi |
|  | PIK3R1 | Phosphoinositide-3-Kinase Regulatory Subunit 1 | 36hpi vs 48 hpi |
|  |  |  | 48hpi vs 60 hpi |
| Th1 and Th2 cell differentiation | CD3D | CD3d Molecule | 0hpi vs 12 hpi |
|  | DLL1 | Delta Like Canonical Notch Ligand 1 | 0hpi vs 12 hpi |
|  | DLL4 | Delta Like Canonical Notch Ligand 4 | 0hpi vs 12 hpi |
|  | FOS | Fos Proto-Oncogene, AP-1 Transcription Factor Subunit | 0hpi vs 12 hpi |
|  | JAG2 | Jagged Canonical Notch Ligand 2 | 0hpi vs 12 hpi |
|  | LCK | LCK Proto-Oncogene, Src Family Tyrosine Kinase | 0hpi vs 12 hpi |
|  | NFKBIE | NFKB Inhibitor Epsilon | 0hpi vs 12 hpi |
|  | DLL4 | Delta Like Canonical Notch Ligand 4 | 12hpi vs 24 hpi |
|  | FOS | Fos Proto-Oncogene, AP-1 Transcription Factor Subunit | 12hpi vs 24 hpi |
|  | IL-12B | Interleukin 12B | 12hpi vs 24 hpi |
|  | JAG1 | Jagged Canonical Notch Ligand 1 | 12hpi vs 24 hpi |
|  | JUN | Jun Proto-Oncogene, AP-1 Transcription Factor Subunit | 12hpi vs 24 hpi |
|  | LCK | LCK Proto-Oncogene, Src Family Tyrosine Kinase | 12hpi vs 24 hpi |
|  | MAPK10 | Mitogen-Activated Protein Kinase 10 | 12hpi vs 24 hpi |
|  | NFATC2 | Nuclear Factor Of Activated T Cells 2 | 12hpi vs 24 hpi |
|  | NFKB1 | Nuclear Factor Kappa B Subunit 1 | 12hpi vs 24 hpi |
|  | NFKBIA | NFKB Inhibitor Alpha | 12hpi vs 24 hpi |
|  | NFKBIE | NFKB Inhibitor Epsilon | 12hpi vs 24 hpi |
|  | NOTCH1 | Notch Receptor 1 | 12hpi vs 24 hpi |
|  | RUNX3 | RUNX Family Transcription Factor 3 | 12hpi vs 24 hpi |
|  | FOS | Fos Proto-Oncogene, AP-1 Transcription Factor Subunit | 24hpi vs 36hpi |
|  | IL-12B | Interleukin 12B | 24hpi vs 36hpi |
|  | IL2RB | Interleukin 2 Receptor Subunit Beta | 24hpi vs 36hpi |
|  | JAG1 | Jagged Canonical Notch Ligand 1 | 24hpi vs 36hpi |
|  | JUN | Jun Proto-Oncogene, AP-1 Transcription Factor Subunit | 24hpi vs 36hpi |
|  | MAF | MAF BZIP Transcription Factor | 24hpi vs 36hpi |
|  | MAPK10 | Mitogen-Activated Protein Kinase 10 | 24hpi vs 36hpi |
|  | NFKB1 | Nuclear Factor Kappa B Subunit 1 | 24hpi vs 36hpi |
|  | NFKBIA， | NFKB Inhibitor Alpha | 24hpi vs 36hpi |
|  | NFKBIE | NFKB Inhibitor Epsilon | 24hpi vs 36hpi |
|  | NOTCH1 | Notch Receptor 1 | 24hpi vs 36hpi |
|  | RBPJ | Recombination Signal Binding Protein For Immunoglobulin Kappa J Region | 24hpi vs 36hpi |
|  | RUNX3 | RUNX Family Transcription Factor 3 | 24hpi vs 36hpi |
|  | STAT5B | Signal Transducer And Activator Of Transcription 5B | 24hpi vs 36hpi |
|  | CD3E | CD3e Molecule | 36hpi vs 48 hpi |
|  | FOS | Fos Proto-Oncogene, AP-1 Transcription Factor Subunit | 36hpi vs 48 hpi |
|  | GATA3 | GATA Binding Protein 3 | 36hpi vs 48 hpi |
|  | IL21R | Interleukin 21 Receptor | 36hpi vs 48 hpi |
|  | IL2RA | Interleukin 2 Receptor Subunit Alpha | 36hpi vs 48 hpi |
|  | JUN | Jun Proto-Oncogene, AP-1 Transcription Factor Subunit | 36hpi vs 48 hpi |
|  | MAPK10 | Mitogen-Activated Protein Kinase 10 | 36hpi vs 48 hpi |
|  | NFATC2 | Nuclear Factor Of Activated T Cells 2 | 36hpi vs 48 hpi |
|  | NFKB1 | Nuclear Factor Kappa B Subunit 1 | 36hpi vs 48 hpi |
|  | NFKBIA | NFKB Inhibitor Alpha | 36hpi vs 48 hpi |
|  | NFKBIE | NFKB Inhibitor Epsilon | 36hpi vs 48 hpi |
|  | RORA | RAR Related Orphan Receptor A | 36hpi vs 48 hpi |
|  | RUNX1 | RUNX Family Transcription Factor 1 | 36hpi vs 48 hpi |
|  | STAT5B | Signal Transducer And Activator Of Transcription 5B | 36hpi vs 48 hpi |
|  | TGFBR2 | Transforming Growth Factor Beta Receptor 2 | 36hpi vs 48 hpi |
|  | AHR | Aryl Hydrocarbon Receptor | 48hpi vs 60 hpi |
|  | FOS | Fos Proto-Oncogene, AP-1 Transcription Factor Subunit | 48hpi vs 60 hpi |
|  | GATA3 | GATA Binding Protein 3 | 48hpi vs 60 hpi |
|  | IL1R1 | Interleukin 1 Receptor Type 1 | 48hpi vs 60 hpi |
|  | IL21R | Interleukin 21 Receptor | 48hpi vs 60 hpi |
|  | IL2RA | Interleukin 2 Receptor Subunit Alpha | 48hpi vs 60 hpi |
|  | IL2RB | Interleukin 2 Receptor Subunit Beta | 48hpi vs 60 hpi |
|  | JUN | Jun Proto-Oncogene, AP-1 Transcription Factor Subunit | 48hpi vs 60 hpi |
|  | MAPK10 | Mitogen-Activated Protein Kinase 10 | 48hpi vs 60 hpi |
|  | MAPK12 | Mitogen-Activated Protein Kinase 12 | 48hpi vs 60 hpi |
|  | NFKB1 | Nuclear Factor Kappa B Subunit 1 | 48hpi vs 60 hpi |
|  | NFKBIA | NFKB Inhibitor Alpha | 48hpi vs 60 hpi |
|  | NFKBIE | NFKB Inhibitor Epsilon | 48hpi vs 60 hpi |
|  | RUNX1 | RUNX Family Transcription Factor 1 | 48hpi vs 60 hpi |
|  | RXRA | Retinoid X Receptor Alpha | 48hpi vs 60 hpi |
|  | RXRG | Retinoid X Receptor Gamma | 48hpi vs 60 hpi |
|  | STAT5B | Signal Transducer And Activator Of Transcription 5B | 48hpi vs 60 hpi |
|  | TGFBR2 | Transforming Growth Factor Beta Receptor 2 | 48hpi vs 60 hpi |
|  |  |  |  |
|  |  |  |  |
