# Supplementary material for: Transcriptome Analysis of Genes Responding to Infection of Leghorn Male Hepatocellular Cells With Fowl Adenovirus Serotype 4
Source: Front Vet Sci. 2022 Jun 14;9:871038. doi: 10.3389/fvets.2022.871038 (PMC9237548; doi:10.3389/fvets.2022.871038)
Supplement: Supplementary file 14 [file Table_8.DOCX]

Differentially expressed gene (DEGs) involved in host metabolism response to FAdV-4 in LMH cells at 12,24,36,48,60hpi .

| Biological process | Gene symbol | Gene description | Source of DEGs |
| --- | --- | --- | --- |
| Carbohydrate metabolism | ATP1B1 | ATPase Na+/K+ Transporting Subunit Beta 1 | 0hpi vs 12 hpi |
|  | PIK3CB | Phosphatidylinositol-4,5-Bisphosphate 3-Kinase Catalytic Subunit Beta | 0hpi vs 12 hpi |
|  | PIK3R1 | Phosphoinositide-3-Kinase Regulatory Subunit 1 | 0hpi vs 12 hpi |
|  | PRKCB | Protein Kinase C Beta | 0hpi vs 12 hpi |
|  | SLC2A2 | Solute Carrier Family 2 Member 2 | 0hpi vs 12 hpi |
|  | G6PC2 | Glucose-6-Phosphatase Catalytic Subunit 2 | 12hpi vs 24 hpi |
|  | LCT | Lactase | 12hpi vs 24 hpi |
|  | PIK3CB | Phosphatidylinositol-4,5-Bisphosphate 3-Kinase Catalytic Subunit Beta | 12hpi vs 24 hpi |
|  | SLC37A4 | Solute Carrier Family 37 Member 4 | 12hpi vs 24 hpi |
|  | CACNA1D | Calcium Voltage-Gated Channel Subunit Alpha1 D | 24hpi vs 36hpi |
|  | SLC2A5 | Solute Carrier Family 2 Member 5 | 24hpi vs 36hpi |
|  | SLC5A1 | Solute Carrier Family 5 Member 1 | 24hpi vs 36hpi |
|  | CACNA1D | Calcium Voltage-Gated Channel Subunit Alpha1 D | 36hpi vs 48 hpi |
|  | PIK3CB | Phosphatidylinositol-4,5-Bisphosphate 3-Kinase Catalytic Subunit Beta | 36hpi vs 48 hpi |
|  | PIK3CD | Phosphatidylinositol-4,5-Bisphosphate 3-Kinase Catalytic Subunit Delta | 36hpi vs 48 hpi |
|  | SLC2A2 | Solute Carrier Family 2 Member 2 | 36hpi vs 48 hpi |
|  | SLC2A5 | Solute Carrier Family 2 Member 5 | 36hpi vs 48 hpi |
|  | SLC37A4 | Solute Carrier Family 37 Member 4 | 36hpi vs 48 hpi |
|  | SLC5A1 | Solute Carrier Family 5 Member 1 | 36hpi vs 48 hpi |
|  | CACNA1D | Calcium Voltage-Gated Channel Subunit Alpha1 D | 48hpi vs 60 hpi |
|  | PIK3CB | Phosphatidylinositol-4,5-Bisphosphate 3-Kinase Catalytic Subunit Beta | 48hpi vs 60 hpi |
|  | PIK3CD | Phosphatidylinositol-4,5-Bisphosphate 3-Kinase Catalytic Subunit Delta | 48hpi vs 60 hpi |
|  | PIK3R1 | Phosphoinositide-3-Kinase Regulatory Subunit 1 | 48hpi vs 60 hpi |
|  | SLC2A5 | Solute Carrier Family 2 Member 5 | 48hpi vs 60 hpi |
|  | SLC5A1 | Solute Carrier Family 5 Member 1 | 48hpi vs 60 hpi |
| Biosynthesis of amino acids | ALDOB | Aldolase, Fructose-Bisphosphate B | 0hpi vs 12 hpi |
|  | BCAT1 | Branched Chain Amino Acid Transaminase 1 | 0hpi vs 12 hpi |
|  | CPS1 | Carbamoyl-Phosphate Synthase 1 | 0hpi vs 12 hpi |
|  | GLUL | Glutamate-Ammonia Ligase | 0hpi vs 12 hpi |
|  | MAT1A | Methionine Adenosyltransferase 1A | 0hpi vs 12 hpi |
|  | PFKM | Phosphofructokinase, Muscle | 0hpi vs 12 hpi |
|  | PFKM | Phosphofructokinase, Muscle | 24hpi vs 36hpi |
|  | PRPS2 | Phosphoribosyl Pyrophosphate Synthetase 2 | 24hpi vs 36hpi |
|  | ALDOB | Aldolase, Fructose-Bisphosphate B | 36hpi vs 48 hpi |
|  | GLUL | Glutamate-Ammonia Ligase | 36hpi vs 48 hpi |
|  | PAH | Phenylalanine Hydroxylase | 36hpi vs 48 hpi |
|  | PFKM | Phosphofructokinase, Muscle | 36hpi vs 48 hpi |
|  | ALDOB | Aldolase, Fructose-Bisphosphate B | 48hpi vs 60 hpi |
|  | ENO2 | Enolase 2 | 48hpi vs 60 hpi |
|  | GLUL | Glutamate-Ammonia Ligase | 48hpi vs 60 hpi |
| Ether lipid metabolism | CHPT1 | Choline Phosphotransferase 1 | 0hpi vs 12 hpi |
|  | ENPP2 | Ectonucleotide Pyrophosphatase/Phosphodiesterase 2 | 0hpi vs 12 hpi |
|  | ENPP6 | Ectonucleotide Pyrophosphatase/Phosphodiesterase 6 | 0hpi vs 12 hpi |
|  | LPCAT2 | Lysophosphatidylcholine Acyltransferase 2 | 0hpi vs 12 hpi |
|  | PLPP3 | Phospholipid Phosphatase 3 | 0hpi vs 12 hpi |
|  | ENPP2 | Ectonucleotide Pyrophosphatase/Phosphodiesterase 2 | 12hpi vs 24 hpi |
|  | PLA2G7 | Phospholipase A2 Group VII | 12hpi vs 24 hpi |
|  | ENPP2 | Ectonucleotide Pyrophosphatase/Phosphodiesterase 2 | 24hpi vs 36hpi |
|  | ENPP6 | Ectonucleotide Pyrophosphatase/Phosphodiesterase 6 | 24hpi vs 36hpi |
|  | GAL3ST1 | Galactose-3-O-Sulfotransferase 1 | 24hpi vs 36hpi |
|  | PLA2G4F | Phospholipase A2 Group IVF | 24hpi vs 36hpi |
|  | PLA2G7 | Phospholipase A2 Group VII | 24hpi vs 36hpi |
|  | ENPP2 | Ectonucleotide Pyrophosphatase/Phosphodiesterase 2 | 36hpi vs 48 hpi |
|  | GAL3ST1 | Galactose-3-O-Sulfotransferase 1 | 36hpi vs 48 hpi |
|  | PLA2G4F | Phospholipase A2 Group IVF | 36hpi vs 48 hpi |
|  | PLA2G7 | Phospholipase A2 Group VII | 36hpi vs 48 hpi |
|  | PLPP3 | Phospholipid Phosphatase 3 | 36hpi vs 48 hpi |
|  | PAFAH2 | Platelet Activating Factor Acetylhydrolase 2 | 48hpi vs 60 hpi |
|  | PLA2G4F | Phospholipase A2 Group IVF | 48hpi vs 60 hpi |
|  | PLPP3 | Phospholipid Phosphatase 3 | 48hpi vs 60 hpi |
| purine metabolism | AK7 | Adenylate Kinase 7 | 0hpi vs 12 hpi |
|  | ENTPD1 | Ectonucleoside Triphosphate Diphosphohydrolase 1 | 0hpi vs 12 hpi |
|  | ENTPD8 | Ectonucleoside Triphosphate Diphosphohydrolase 8 | 0hpi vs 12 hpi |
|  | PDE1A | Phosphodiesterase 1A | 0hpi vs 12 hpi |
|  | PDE1B | Phosphodiesterase 1B | 0hpi vs 12 hpi |
|  | ADCY3 | Adenylate Cyclase 3 | 12hpi vs 24 hpi |
|  | AMPD1 | Adenosine Monophosphate Deaminase 1 | 12hpi vs 24 hpi |
|  | ENTPD3 | Ectonucleoside Triphosphate Diphosphohydrolase 3 | 12hpi vs 24 hpi |
|  | GDA | Guanine Deaminase | 12hpi vs 24 hpi |
|  | NME4 | NME/NM23 Nucleoside Diphosphate Kinase 4 | 12hpi vs 24 hpi |
|  | NT5C1A | 5'-Nucleotidase, Cytosolic IA | 12hpi vs 24 hpi |
|  | NUDT2 | Nudix Hydrolase 2 | 12hpi vs 24 hpi |
|  | NUDT5 | Nudix Hydrolase 5 | 12hpi vs 24 hpi |
|  | PAPSS2 | 3'-Phosphoadenosine 5'-Phosphosulfate Synthase 2 | 12hpi vs 24 hpi |
|  | PDE1B | Phosphodiesterase 1B | 12hpi vs 24 hpi |
|  | PDE1C | Phosphodiesterase 1C | 12hpi vs 24 hpi |
|  | PDE4B | Phosphodiesterase 4B | 12hpi vs 24 hpi |
|  | PDE4D | Phosphodiesterase 4D | 12hpi vs 24 hpi |
|  | PDE5A | Phosphodiesterase 5A | 12hpi vs 24 hpi |
|  | PDE6G | Phosphodiesterase 6G | 12hpi vs 24 hpi |
|  | POLR2F | RNA Polymerase II, I And III Subunit F | 12hpi vs 24 hpi |
|  | ADCY3 | Adenylate Cyclase 3 | 24hpi vs 36hpi |
|  | AK4 | Adenylate Kinase 4 | 24hpi vs 36hpi |
|  | AK6 | Adenylate Kinase 6 | 24hpi vs 36hpi |
|  | ENTPD3 | Ectonucleoside Triphosphate Diphosphohydrolase 3 | 24hpi vs 36hpi |
|  | GDA | Guanine Deaminase | 24hpi vs 36hpi |
|  | GUCY2C | Guanylate Cyclase 2C | 24hpi vs 36hpi |
|  | NT5C1A | 5'-Nucleotidase, Cytosolic IA | 24hpi vs 36hpi |
|  | PAPSS2 | 3'-Phosphoadenosine 5'-Phosphosulfate Synthase 2 | 24hpi vs 36hpi |
|  | PDE1C | Phosphodiesterase 1C | 24hpi vs 36hpi |
|  | PDE4B | Phosphodiesterase 4B | 24hpi vs 36hpi |
|  | PDE4D | Phosphodiesterase 4D | 24hpi vs 36hpi |
|  | PDE5A | Phosphodiesterase 5A | 24hpi vs 36hpi |
|  | PDE7B | Phosphodiesterase 7B | 24hpi vs 36hpi |
|  | PDE9A | Phosphodiesterase 9A | 24hpi vs 36hpi |
|  | PRPS2 | Phosphoribosyl Pyrophosphate Synthetase 2 | 24hpi vs 36hpi |
|  | ADCY2 | Adenylate Cyclase 2 | 36hpi vs 48 hpi |
|  | ADCY8 | Adenylate Cyclase 8 | 36hpi vs 48 hpi |
|  | AK6 | Adenylate Kinase 6 | 36hpi vs 48 hpi |
|  | AK7 | Adenylate Kinase 7 | 36hpi vs 48 hpi |
|  | AMPD1 | Adenosine Monophosphate Deaminase 1 | 36hpi vs 48 hpi |
|  | ENTPD3 | Ectonucleoside Triphosphate Diphosphohydrolase 3 | 36hpi vs 48 hpi |
|  | GDA | Guanine Deaminase | 36hpi vs 48 hpi |
|  | NT5C1A | 5'-Nucleotidase, Cytosolic IA | 36hpi vs 48 hpi |
|  | NUDT2 | Nudix Hydrolase 2 | 36hpi vs 48 hpi |
|  | NUDT5 | Nudix Hydrolase 5 | 36hpi vs 48 hpi |
|  | PAPSS2 | 3'-Phosphoadenosine 5'-Phosphosulfate Synthase 2 | 36hpi vs 48 hpi |
|  | PDE1C | Phosphodiesterase 1C | 36hpi vs 48 hpi |
|  | PDE4B | Phosphodiesterase 4B | 36hpi vs 48 hpi |
|  | PDE4D | Phosphodiesterase 4D | 36hpi vs 48 hpi |
|  | PDE5A | Phosphodiesterase 5A | 36hpi vs 48 hpi |
|  | PDE7B | Phosphodiesterase 7B | 36hpi vs 48 hpi |
|  | ADCY7 | Adenylate Cyclase 7 | 48hpi vs 60 hpi |
|  | ADCY8 | Adenylate Cyclase 8 | 48hpi vs 60 hpi |
|  | AK6 | Adenylate Kinase 6 | 48hpi vs 60 hpi |
|  | AMPD1 | Adenosine Monophosphate Deaminase 1 | 48hpi vs 60 hpi |
|  | ENTPD3 | Ectonucleoside Triphosphate Diphosphohydrolase 3 | 48hpi vs 60 hpi |
|  | GUCY1A2 | Guanylate Cyclase 1 Soluble Subunit Alpha 2 | 48hpi vs 60 hpi |
|  | GUCY2C, | Guanylate Cyclase 2C | 48hpi vs 60 hpi |
|  | NT5C1A | 5'-Nucleotidase, Cytosolic IA | 48hpi vs 60 hpi |
|  | NT5DC4 | 5'-Nucleotidase Domain Containing 4 | 48hpi vs 60 hpi |
|  | PAPSS2 | 3'-Phosphoadenosine 5'-Phosphosulfate Synthase 2 | 48hpi vs 60 hpi |
|  | PDE10A | Phosphodiesterase 10A | 48hpi vs 60 hpi |
|  | PDE3A | Phosphodiesterase 3A | 48hpi vs 60 hpi |
|  | PDE4B | Phosphodiesterase 4B | 48hpi vs 60 hpi |
|  | PDE4D | Phosphodiesterase 4D | 48hpi vs 60 hpi |
|  | PDE5A | Phosphodiesterase 5A | 48hpi vs 60 hpi |
|  | PDE6G | Phosphodiesterase 6G | 48hpi vs 60 hpi |
|  | PDE7B | Phosphodiesterase 7B | 48hpi vs 60 hpi |
|  | PDE9A | Phosphodiesterase 9A | 48hpi vs 60 hpi |
| Pyrimidine metabolism | DPYS | Dihydropyrimidinase | 0hpi vs 12 hpi |
|  | ENTPD3 | Ectonucleoside Triphosphate Diphosphohydrolase 3 | 0hpi vs 12 hpi |
|  | NT5C1A | 5'-Nucleotidase, Cytosolic IA | 0hpi vs 12 hpi |
|  | NT5DC4 | 5'-Nucleotidase Domain Containing 4 | 0hpi vs 12 hpi |
|  | DPYS | Dihydropyrimidinase | 12hpi vs 24 hpi |
|  | ENTPD3 | Ectonucleoside Triphosphate Diphosphohydrolase 3 | 12hpi vs 24 hpi |
|  | NME4 | NME/NM23 Nucleoside Diphosphate Kinase 4 | 12hpi vs 24 hpi |
|  | NT5C1A | 5'-Nucleotidase, Cytosolic IA | 12hpi vs 24 hpi |
|  | NUDT2 | Nudix Hydrolase 2 | 12hpi vs 24 hpi |
|  | POLR2F | RNA Polymerase II, I And III Subunit F | 12hpi vs 24 hpi |
|  | CMPK2 | Cytidine/Uridine Monophosphate Kinase 2 | 24hpi vs 36hpi |
|  | ENTPD3 | Ectonucleoside Triphosphate Diphosphohydrolase 3 | 24hpi vs 36hpi |
|  | NT5C1A | 5'-Nucleotidase, Cytosolic IA | 24hpi vs 36hpi |
|  | CMPK2 | Cytidine/Uridine Monophosphate Kinase 2 | 36hpi vs 48 hpi |
|  | ENTPD3 | Ectonucleoside Triphosphate Diphosphohydrolase 3 | 36hpi vs 48 hpi |
|  | NT5C1A | 5'-Nucleotidase, Cytosolic IA | 36hpi vs 48 hpi |
|  | NUDT2 | Nudix Hydrolase 2 | 36hpi vs 48 hpi |
|  | TXNRD1 | Thioredoxin Reductase 1 | 36hpi vs 48 hpi |
|  | DPYS | Dihydropyrimidinase | 48hpi vs 60 hpi |
|  | ENTPD3 | Ectonucleoside Triphosphate Diphosphohydrolase 3 | 48hpi vs 60 hpi |
|  | NT5C1A | 5'-Nucleotidase, Cytosolic IA | 48hpi vs 60 hpi |
|  | NT5DC4 | 5'-Nucleotidase Domain Containing 4 | 48hpi vs 60 hpi |
|  | CA6 | Carbonic Anhydrase 6 | 0hpi vs 12 hpi |
|  | CPS1 | Carbamoyl-Phosphate Synthase 1 | 0hpi vs 12 hpi |
|  | GLUL | Glutamate-Ammonia Ligase | 0hpi vs 12 hpi |
|  | CA6 | Carbonic Anhydrase 6 | 12hpi vs 24 hpi |
|  | CA2 | Carbonic Anhydrase 2 | 24hpi vs 36hpi |
|  | CA2 | Carbonic Anhydrase 2 | 36hpi vs 48 hpi |
|  | GLUL | Glutamate-Ammonia Ligase | 36hpi vs 48 hpi |
|  | CA2 | Carbonic Anhydrase 2 | 48hpi vs 60 hpi |
|  | CA8 | Carbonic Anhydrase 8 | 48hpi vs 60 hpi |
|  | GLUL | Glutamate-Ammonia Ligase | 48hpi vs 60 hpi |
| Biosynthesis of secondary metabolites | ACAT2 | Acetyl-CoA Acetyltransferase 2 | 0hpi vs 12 hpi |
|  | ADH1C | Alcohol Dehydrogenase 1C (Class I), Gamma Polypeptide | 0hpi vs 12 hpi |
|  | AGPAT2 | 1-Acylglycerol-3-Phosphate O-Acyltransferase 2 | 0hpi vs 12 hpi |
|  | AK7 | Adenylate Kinase 7 | 0hpi vs 12 hpi |
|  | ALAS2 | 5'-Aminolevulinate Synthase 2 | 0hpi vs 12 hpi |
|  | ALDOB | Aldolase, Fructose-Bisphosphate B | 0hpi vs 12 hpi |
|  | BCAT1 | Branched Chain Amino Acid Transaminase 1 | 0hpi vs 12 hpi |
|  | COQ2 | Coenzyme Q2, Polyprenyltransferase | 0hpi vs 12 hpi |
|  | CYP51A1 | Cytochrome P450 Family 51 Subfamily A Member 1 | 0hpi vs 12 hpi |
|  | DGKI | Diacylglycerol Kinase Iota | 0hpi vs 12 hpi |
|  | DHCR24 | 24-Dehydrocholesterol Reductase | 0hpi vs 12 hpi |
|  | DHCR7 | 7-Dehydrocholesterol Reductase | 0hpi vs 12 hpi |
|  | FADS2 | Fatty Acid Desaturase 2 | 0hpi vs 12 hpi |
|  | GAD1 | Glutamate Decarboxylase 1 | 0hpi vs 12 hpi |
|  | GADL1 | Glutamate Decarboxylase Like 1 | 0hpi vs 12 hpi |
|  | GPAM | Glycerol-3-Phosphate Acyltransferase, Mitochondrial | 0hpi vs 12 hpi |
|  | GPAT3 | Glycerol-3-Phosphate Acyltransferase 3 | 0hpi vs 12 hpi |
|  | H6PD | Hexose-6-Phosphate Dehydrogenase/Glucose 1-Dehydrogenase | 0hpi vs 12 hpi |
|  | HMGCR | 3-Hydroxy-3-Methylglutaryl-CoA Reductase | 0hpi vs 12 hpi |
|  | HMGCS1 | 3-Hydroxy-3-Methylglutaryl-CoA Synthase 1 | 0hpi vs 12 hpi |
|  | HMOX1 | Heme Oxygenase 1 | 0hpi vs 12 hpi |
|  | LPIN1 | Lipin 1 | 0hpi vs 12 hpi |
|  | MAT1A | Methionine Adenosyltransferase 1A | 0hpi vs 12 hpi |
|  | ODC1 | Ornithine Decarboxylase 1 | 0hpi vs 12 hpi |
|  | PCK1 | Phosphoenolpyruvate Carboxykinase 1 | 0hpi vs 12 hpi |
|  | PFKM | Phosphofructokinase, Muscle | 0hpi vs 12 hpi |
|  | PLPP3 | Phospholipid Phosphatase 3 | 0hpi vs 12 hpi |
|  | ACOX3 | Acyl-CoA Oxidase 3, Pristanoyl | 12hpi vs 24 hpi |
|  | ADH1C | Alcohol Dehydrogenase 1C (Class I), Gamma Polypeptide | 12hpi vs 24 hpi |
|  | ALAS2 | 5'-Aminolevulinate Synthase 2 | 12hpi vs 24 hpi |
|  | AMPD1 | Adenosine Monophosphate Deaminase 1 | 12hpi vs 24 hpi |
|  | DGKG | Diacylglycerol Kinase Gamma | 12hpi vs 24 hpi |
|  | DHCR24 | 24-Dehydrocholesterol Reductase | 12hpi vs 24 hpi |
|  | ELOVL2 | ELOVL Fatty Acid Elongase 2 | 12hpi vs 24 hpi |
|  | GAD2 | Glutamate Decarboxylase 2 | 12hpi vs 24 hpi |
|  | HDC | Histidine Decarboxylase | 12hpi vs 24 hpi |
|  | HMGCS2 | 3-Hydroxy-3-Methylglutaryl-CoA Synthase 2 | 12hpi vs 24 hpi |
|  | IDNK | IDNK Gluconokinase | 12hpi vs 24 hpi |
|  | NME4 | NME/NM23 Nucleoside Diphosphate Kinase 4 | 12hpi vs 24 hpi |
|  | NT5C1A | 5'-Nucleotidase, Cytosolic IA | 12hpi vs 24 hpi |
|  | PCK1 | Phosphoenolpyruvate Carboxykinase 1 | 12hpi vs 24 hpi |
|  | PLA2G7 | Phospholipase A2 Group VII | 12hpi vs 24 hpi |
|  | SQLE | Squalene Epoxidase | 12hpi vs 24 hpi |
|  | UGT1A1 | UDP Glucuronosyltransferase Family 1 Member A1 | 12hpi vs 24 hpi |
|  | UROS | Uroporphyrinogen III Synthase | 12hpi vs 24 hpi |
|  | VKORC1L1 | Vitamin K Epoxide Reductase Complex Subunit 1 Like 1 | 12hpi vs 24 hpi |
|  | ACOX3 | Acyl-CoA Oxidase 3, Pristanoyl | 24hpi vs 36hpi |
|  | AK4 | Adenylate Kinase 4 | 24hpi vs 36hpi |
|  | AK6 | Adenylate Kinase 6 | 24hpi vs 36hpi |
|  | ALAS2 | 5'-Aminolevulinate Synthase 2 | 24hpi vs 36hpi |
|  | BLVRA | Biliverdin Reductase A | 24hpi vs 36hpi |
|  | CYP51A1 | Cytochrome P450 Family 51 Subfamily A Member 1 | 24hpi vs 36hpi |
|  | DHCR24 | 24-Dehydrocholesterol Reductase | 24hpi vs 36hpi |
|  | DHCR7 | 7-Dehydrocholesterol Reductase | 24hpi vs 36hpi |
|  | FADS2 | Fatty Acid Desaturase 2 | 24hpi vs 36hpi |
|  | FDFT1 | Farnesyl-Diphosphate Farnesyltransferase 1 | 24hpi vs 36hpi |
|  | FDPS | Farnesyl Diphosphate Synthase | 24hpi vs 36hpi |
|  | GADL1 | Glutamate Decarboxylase Like 1 | 24hpi vs 36hpi |
|  | HMGCS1 | 3-Hydroxy-3-Methylglutaryl-CoA Synthase 1 | 24hpi vs 36hpi |
|  | HMOX1 | Heme Oxygenase 1 | 24hpi vs 36hpi |
|  | HSD17B12 | Hydroxysteroid 17-Beta Dehydrogenase 12 | 24hpi vs 36hpi |
|  | IDNK, | IDNK Gluconokinase | 24hpi vs 36hpi |
|  | LDHA | Lactate Dehydrogenase A | 24hpi vs 36hpi |
|  | LPIN1 | Lipin 1 | 24hpi vs 36hpi |
|  | NOS2 | Nitric Oxide Synthase 2 | 24hpi vs 36hpi |
|  | NT5C1A | 5'-Nucleotidase, Cytosolic IA | 24hpi vs 36hpi |
|  | PCK1 | Phosphoenolpyruvate Carboxykinase 1 | 24hpi vs 36hpi |
|  | PFKM | Phosphofructokinase, Muscle | 24hpi vs 36hpi |
|  | PGP | Phosphoglycolate Phosphatase | 24hpi vs 36hpi |
|  | PLA2G4F | Phospholipase A2 Group IVF | 24hpi vs 36hpi |
|  | PLA2G7 | Phospholipase A2 Group VII | 24hpi vs 36hpi |
|  | PRPS2 | Phosphoribosyl Pyrophosphate Synthetase 2 | 24hpi vs 36hpi |
|  | SC5D | Sterol-C5-Desaturase | 24hpi vs 36hpi |
|  | SQLE | Squalene Epoxidase | 24hpi vs 36hpi |
|  | TRIT1 | TRNA Isopentenyltransferase 1 | 24hpi vs 36hpi |
|  | VKORC1L1 | Vitamin K Epoxide Reductase Complex Subunit 1 Like 1 | 24hpi vs 36hpi |
|  | ACOX3 | Acyl-CoA Oxidase 3, Pristanoyl | 36hpi vs 48 hpi |
|  | AK6 | Adenylate Kinase 6 | 36hpi vs 48 hpi |
|  | AK7 | Adenylate Kinase 7 | 36hpi vs 48 hpi |
|  | ALDOB | Aldolase, Fructose-Bisphosphate B | 36hpi vs 48 hpi |
|  | AMPD1 | Adenosine Monophosphate Deaminase 1 | 36hpi vs 48 hpi |
|  | BLVRA | Biliverdin Reductase A | 36hpi vs 48 hpi |
|  | CYP51A1 | Cytochrome P450 Family 51 Subfamily A Member 1 | 36hpi vs 48 hpi |
|  | DHCR24 | 24-Dehydrocholesterol Reductase | 36hpi vs 48 hpi |
|  | FADS2 | Fatty Acid Desaturase 2 | 36hpi vs 48 hpi |
|  | FBP1 | Fructose-Bisphosphatase 1 | 36hpi vs 48 hpi |
|  | FDPS | Farnesyl Diphosphate Synthase | 36hpi vs 48 hpi |
|  | GADL1 | Glutamate Decarboxylase Like 1 | 36hpi vs 48 hpi |
|  | HMGCS1 | 3-Hydroxy-3-Methylglutaryl-CoA Synthase 1 | 36hpi vs 48 hpi |
|  | HMGCS2 | 3-Hydroxy-3-Methylglutaryl-CoA Synthase 2 | 36hpi vs 48 hpi |
|  | HMOX1 | Heme Oxygenase 1 | 36hpi vs 48 hpi |
|  | IDNK, | IDNK Gluconokinase | 36hpi vs 48 hpi |
|  | NT5C1A | 5'-Nucleotidase, Cytosolic IA | 36hpi vs 48 hpi |
|  | PCK1 | Phosphoenolpyruvate Carboxykinase 1 | 36hpi vs 48 hpi |
|  | PFKM | Phosphofructokinase, Muscle | 36hpi vs 48 hpi |
|  | PLA2G4F | Phospholipase A2 Group IVF | 36hpi vs 48 hpi |
|  | PLA2G7 | Phospholipase A2 Group VII | 36hpi vs 48 hpi |
|  | PLPP3 | Phospholipid Phosphatase 3 | 36hpi vs 48 hpi |
|  | RGN | Regucalcin | 36hpi vs 48 hpi |
|  | SC5D | Sterol-C5-Desaturase | 36hpi vs 48 hpi |
|  | SQLE | Squalene Epoxidase | 36hpi vs 48 hpi |
|  | TRIT1 | TRNA Isopentenyltransferase 1 | 36hpi vs 48 hpi |
|  | VKORC1L1 | Vitamin K Epoxide Reductase Complex Subunit 1 Like 1 | 36hpi vs 48 hpi |
|  | ACOX3 | Acyl-CoA Oxidase 3, Pristanoyl | 48hpi vs 60 hpi |
|  | ADH6 | Alcohol Dehydrogenase 6 (Class V) | 48hpi vs 60 hpi |
|  | AK6 | Adenylate Kinase 6 | 48hpi vs 60 hpi |
|  | ALAS2 | 5'-Aminolevulinate Synthase 2 | 48hpi vs 60 hpi |
|  | ALDOB | Aldolase, Fructose-Bisphosphate B | 48hpi vs 60 hpi |
|  | AMPD1 | Adenosine Monophosphate Deaminase 1 | 48hpi vs 60 hpi |
|  | DGKD | Diacylglycerol Kinase Delta | 48hpi vs 60 hpi |
|  | DGKI | Diacylglycerol Kinase Iota | 48hpi vs 60 hpi |
|  | ENO2 | Enolase 2 | 48hpi vs 60 hpi |
|  | FLAD1 | Flavin Adenine Dinucleotide Synthetase 1 | 48hpi vs 60 hpi |
|  | GLDC | Glycine Decarboxylase | 48hpi vs 60 hpi |
|  | HAO1 | Hydroxyacid Oxidase 1 | 48hpi vs 60 hpi |
|  | HDC | Histidine Decarboxylase | 48hpi vs 60 hpi |
|  | LPIN1 | Lipin 1 | 48hpi vs 60 hpi |
|  | NOS2 | Nitric Oxide Synthase 2 | 48hpi vs 60 hpi |
|  | NT5C1A | 5'-Nucleotidase, Cytosolic IA | 48hpi vs 60 hpi |
|  | NT5DC4 | 5'-Nucleotidase Domain Containing 4 | 48hpi vs 60 hpi |
|  | PAFAH2, | Platelet Activating Factor Acetylhydrolase 2 | 48hpi vs 60 hpi |
|  | PCK1 | Phosphoenolpyruvate Carboxykinase 1 | 48hpi vs 60 hpi |
|  | PLA2G4F | Phospholipase A2 Group IVF | 48hpi vs 60 hpi |
|  | PLPP3 | Phospholipid Phosphatase 3 | 48hpi vs 60 hpi |
|  | SC5D | Sterol-C5-Desaturase | 48hpi vs 60 hpi |
|  | VKORC1L1 | Vitamin K Epoxide Reductase Complex Subunit 1 Like 1 | 48hpi vs 60 hpi |
| Sphingolipid metabolism | CERS3 | Ceramide Synthase 3 | 0hpi vs 12 hpi |
|  | DEGS1 | Delta 4-Desaturase, Sphingolipid 1 | 0hpi vs 12 hpi |
|  | NEU2 | Neuraminidase 2 | 0hpi vs 12 hpi |
|  | NEU3 | Neuraminidase 3 | 0hpi vs 12 hpi |
|  | SPTLC3 | Serine Palmitoyltransferase Long Chain Base Subunit 3 | 0hpi vs 12 hpi |
|  | CERS3 | Ceramide Synthase 3 | 12hpi vs 24 hp |
|  | DEGS1 | Delta 4-Desaturase, Sphingolipid 1 | 12hpi vs 24 hp |
|  | DEGS2 | Delta 4-Desaturase, Sphingolipid 2 | 12hpi vs 24 hp |
|  | GAL3ST1 | Galactose-3-O-Sulfotransferase 1 | 12hpi vs 24 hp |
|  | LINS1 | Lines Homolog 1 | 12hpi vs 24 hp |
|  | NEU2 | Neuraminidase 2 | 12hpi vs 24 hp |
|  | NEU3 | Neuraminidase 3 | 12hpi vs 24 hp |
|  | SPTLC3 | Serine Palmitoyltransferase Long Chain Base Subunit 3 | 12hpi vs 24 hp |
|  | CERS3 | Ceramide Synthase 3 | 24hpi vs 36hpi |
|  | DEGS1 | Delta 4-Desaturase, Sphingolipid 1 | 24hpi vs 36hpi |
|  | DEGS2 | Delta 4-Desaturase, Sphingolipid 2 | 24hpi vs 36hpi |
|  | GAL3ST1 | Galactose-3-O-Sulfotransferase 1 | 24hpi vs 36hpi |
|  | LINS1 | Lines Homolog 1 | 24hpi vs 36hpi |
|  | NEU2 | Neuraminidase 2 | 24hpi vs 36hpi |
|  | NEU3 | Neuraminidase 3 | 24hpi vs 36hpi |
|  | SPTLC3 | Serine Palmitoyltransferase Long Chain Base Subunit 3 | 24hpi vs 36hpi |
|  | CERS3 | Ceramide Synthase 3 | 36hpi vs 48 hpi |
|  | DEGS1 | Delta 4-Desaturase, Sphingolipid 1 | 36hpi vs 48 hpi |
|  | GAL3ST1 | Galactose-3-O-Sulfotransferase 1 | 36hpi vs 48 hpi |
|  | LINS1 | Lines Homolog 1 | 36hpi vs 48 hpi |
|  | NEU3 | Neuraminidase 3 | 36hpi vs 48 hpi |
|  | PLPP3 | Phospholipid Phosphatase 3 | 36hpi vs 48 hpi |
|  | SGPL1 | Sphingosine-1-Phosphate Lyase 1 | 36hpi vs 48 hpi |
|  | CERS3 | Ceramide Synthase 3 | 48hpi vs 60 hpi |
|  | DEGS1 | Delta 4-Desaturase, Sphingolipid 1 | 48hpi vs 60 hpi |
|  | DEGS2 | Delta 4-Desaturase, Sphingolipid 2 | 48hpi vs 60 hpi |
|  | PLPP3 | Phospholipid Phosphatase 3 | 48hpi vs 60 hpi |
|  | UGCG | UDP-Glucose Ceramide Glucosyltransferase | 48hpi vs 60 hpi |
| Steroid biosynthesis | CYP51A1 | Cytochrome P450 Family 51 Subfamily A Member 1 | 0hpi vs 12 hpi |
|  | DHCR24 | 24-Dehydrocholesterol Reductase | 0hpi vs 12 hpi |
|  | DHCR7 | 7-Dehydrocholesterol Reductase | 0hpi vs 12 hpi |
|  | DHCR24 | 24-Dehydrocholesterol Reductase | 12hpi vs 24 hpi |
|  | MSMO1 | Methylsterol Monooxygenase 1 | 12hpi vs 24 hpi |
|  | SQLE | Squalene Epoxidase | 12hpi vs 24 hpi |
|  | CYP51A1 | Cytochrome P450 Family 51 Subfamily A Member 1 | 24hpi vs 36hpi |
|  | DHCR24 | 24-Dehydrocholesterol Reductase | 24hpi vs 36hpi |
|  | DHCR7 | 7-Dehydrocholesterol Reductase | 24hpi vs 36hpi |
|  | FDFT1 | Farnesyl-Diphosphate Farnesyltransferase 1 | 24hpi vs 36hpi |
|  | MSMO1 | Methylsterol Monooxygenase 1 | 24hpi vs 36hpi |
|  | NSDHL | NAD(P) Dependent Steroid Dehydrogenase-Like | 24hpi vs 36hpi |
|  | SC5D | Sterol-C5-Desaturase | 24hpi vs 36hpi |
|  | SQLE | Squalene Epoxidase | 24hpi vs 36hpi |
|  | CYP51A1 | Cytochrome P450 Family 51 Subfamily A Member 1 | 36hpi vs 48 hpi |
|  | DHCR24 | 24-Dehydrocholesterol Reductase | 36hpi vs 48 hpi |
|  | MSMO1 | Methylsterol Monooxygenase 1 | 36hpi vs 48 hpi |
|  | NSDHL | NAD(P) Dependent Steroid Dehydrogenase-Like | 36hpi vs 48 hpi |
|  | SC5D | Sterol-C5-Desaturase | 36hpi vs 48 hpi |
|  | SQLE | Squalene Epoxidase | 36hpi vs 48 hpi |
| Biosynthesis of amino acids | ALDOB | Aldolase, Fructose-Bisphosphate B | 0hpi vs 12 hpi |
|  | BCAT1 | Branched Chain Amino Acid Transaminase 1 | 0hpi vs 12 hpi |
|  | CPS1 | Carbamoyl-Phosphate Synthase 1 | 0hpi vs 12 hpi |
|  | GLUL | Glutamate-Ammonia Ligase | 0hpi vs 12 hpi |
|  | MAT1A | Methionine Adenosyltransferase 1A | 0hpi vs 12 hpi |
|  | PFKM | Phosphofructokinase, Muscle | 0hpi vs 12 hpi |
|  | PFKM | Phosphofructokinase, Muscle | 24hpi vs 36hpi |
|  | PRPS2 | Phosphoribosyl Pyrophosphate Synthetase 2 | 24hpi vs 36hpi |
|  | ALDOB | Aldolase, Fructose-Bisphosphate B | 36hpi vs 48 hpi |
|  | GLUL | Glutamate-Ammonia Ligase | 36hpi vs 48 hpi |
|  | PAH | Phenylalanine Hydroxylase | 36hpi vs 48 hpi |
|  | PFKM | Phosphofructokinase, Muscle | 36hpi vs 48 hpi |
|  | ALDOB | Aldolase, Fructose-Bisphosphate B | 48hpi vs 60 hpi |
|  | ENO2 | Enolase 2 | 48hpi vs 60 hpi |
|  | GLUL | Glutamate-Ammonia Ligase | 48hpi vs 60 hpi |
