# Supplementary material for: Transcriptome Analysis of Genes Responding to Infection of Leghorn Male Hepatocellular Cells With Fowl Adenovirus Serotype 4
Source: Front Vet Sci. 2022 Jun 14;9:871038. doi: 10.3389/fvets.2022.871038 (PMC9237548; doi:10.3389/fvets.2022.871038)
Supplement: Supplementary file 15 [file Table_9.DOCX]

Primers sequence of the differentially expressed genes for real-time PCR analysis

| Gene | Forward primer | Reverse primer |
| --- | --- | --- |
| EGR1 | TCCACCTCCAGCTTATCCGA | CATCGGACCGTGAAAATCGC |
| SOCS1 | ACCCCAAACGCACCTACTAC | AGCACAACTCCAGCACAGTT |
| SOCS3 | AGACCCGCGATGTCTACTTG | TCAAAGGTCTCCTTGCTGCC |
| IL8L1 | CGTTCAGCGATTGAACTCCG | CTGAATGGCGTTGTCTCCCA |
| BCL2L1 | GACAGCGTCCTCAATGGGAG | AGTGGAACCCGAGTTGAGA |
| CCL4 | TCACAAAGAAGGAGCGCGAA | CAAACAGCACCTGCCATGAG |
| TNFRSF21 | CTGGCAAGTACCTCCACCTG | AGCTTCGTGGTCTCCCCAT |
| CD30 | GCTAAGAACACCTGCATGCG | TGCCAAAATGACAGCATGCC |
| PCK1 | GCCATCAGCTAAAGGGAGC | AAAGGAGATCCAATCGGCCC |
